# Viridicatin from the Antarctic Fungus *Penicillium* sp. Protects Human Microglia and Patient-Derived Peripheral Immune Cells Against Oxidative Stress

**DOI:** 10.3390/antiox15091089

**Published:** 2026-08-30

**Authors:** Cristian Paz, Muhammad Javid Iqbal, Andrea Cristina Paula Lima, Alejandro Luarte, Pablo Lazcano, Ursula Wyneken, María Isabel Behrens, Daniela Ponce, Nicole Jeraldo, Sigisfredo Garnica, Cecilia Villegas, Vaderament-A. Nchiozem-Ngnitedem, Bernd Schmidt, Eric Sperlich, Nicole Cortez, Viviana Burgos

**Affiliations:** 1 Laboratory of Natural Products & Drug Discovery, Department of Basic Sciences, Faculty of Medicine, Universidad de La Frontera, Temuco 4780000, Chile; cristian.paz@ufrontera.cl (C.P.); m.iqbal01@ufromail.cl (M.J.I.); 2 Biomedical Neuroscience Institute, Faculty of Medicine, University of Chile, Santiago 8380453, Chile; acpaulalima@u.uchile.cl; 3 Institute for Research in Dental Sciences, Faculty of Dentistry, University of Chile, Santiago 8380453, Chile; 4 Faculty of Medicine, Universidad de los Andes, Santiago 7620001, Chile; aluarte@uandes.cl (A.L.); uwyneken@uandes.cl (U.W.); 5 Program in Neuroscience, Center for Biomedical Research, and Innovation (CiiB), Universidad de los Andes, Santiago 7620001, Chile; 6 IMPACT—Center of Interventional Medicine for Precision and Advanced Cellular Therapy, Santiago 7620001, Chile; plazcano@uandes.cl; 7 Center for Advanced Clinical Research (CICA), Clinical Hospital of the University of Chile, Santiago 8380447, Chile; behrensl@uchile.cl (M.I.B.); dponcedelavega@gmail.com (D.P.); nicole.jeraldo@ug.uchile.cl (N.J.); 8 Department of Neuroscience, Faculty of Medicine, University of Chile, Santiago 8380453, Chile; 9 Department of Neurology and Neurosurgery, Clinical Hospital of the University of Chile, Santiago 8380447, Chile; 10 Department of Neurology and Psychiatry, German Clinic of Santiago-University of Development, Santiago 7650568, Chile; 11 Laboratorio de Micología, Instituto de Bioquímica y Microbiología, Universidad Austral de Chile, Valdivia 5090000, Chile; sigisfredo.garnica@uach.cl; 12 Departamento de Ciencias Biológicas y Químicas, Facultad de Recursos Naturales, Universidad Católica de Temuco, Temuco 4780000, Chile; cecilia.villegas@uct.cl; 13 Institut für Chemie, Universität Potsdam, Karl-Liebknecht-Str. 24-25, D-14476 Potsdam, Germany; vaderament-alexe.nchiozem-ngnitedem@uni-potsdam.de (V.-A.N.-N.); bernd.schmidt@uni-potsdam.de (B.S.); eric.sperlich@uni-potsdam.de (E.S.); 14 Escuela de Tecnología Médica, Facultad de Salud, Universidad Santo Tomás, Temuco 4780000, Chile; 15 Centro de Investigación en Prevención y Cuidados de la Salud (+SALUD), Facultad de Salud, Universidad Santo Tomás, Temuco 4780000, Chile

**Keywords:** viridicatin, Antarctic fungus, cell protection, oxidative stress, Nrf2/KEAP1 pathway, microglia, in silico

## Abstract

Neurodegenerative diseases remain a major therapeutic challenge, with oxidative stress playing a central role in central and peripheral immune system dysfunction that leads to neuronal loss. Natural products from extreme environments represent an underexplored source of neuroprotective agents. In this study, viridicatin, a quinoline-derived alkaloid, was isolated from the Antarctic fungus *Penicillium* sp. collected from sediments taken from Deception Island, and its structure was unambiguously confirmed by 1D/2D-NMR spectroscopy and single-crystal X-ray diffraction. Viridicatin (100 µM) significantly attenuated H_2_O_2_-induced cytotoxicity in HMC-3 human microglial cells, preserving cell viability and mitochondrial membrane potential. Viridicatin modulated the Nrf2 antioxidant signaling pathway, accompanied by increased expression of the downstream antioxidant enzymes HO-1 and NQO1. Moreover, molecular docking revealed preferential binding to the KEAP1 Kelch domain (−8.0 kcal/mol), suggesting indirect Nrf2 pathway modulation. A 100 ns molecular dynamics simulation with MM-GBSA analysis supported the stability of the viridicatin–KEAP1 complex. Notably, viridicatin rescued peripheral immune cells, i.e., peripheral blood mononuclear cells (PBMCs) obtained from older adults with mild cognitive impairment from H_2_O_2_-induced cell death, bridging the gap between in vitro mechanistic evidence and clinically relevant human cellular models. This is the first report of neuroprotective activity for viridicatin, positioning this Antarctic-derived alkaloid as a compelling candidate for further preclinical development against age-related neurodegeneration.

## 1. Introduction

Neurodegenerative diseases (NDs), including Alzheimer’s, Parkinson’s, and Huntington’s disease, as well as amyotrophic lateral sclerosis (ALS), are a diverse group of disorders causing progressive neuron loss in the central and peripheral nervous systems, leading to neuronal dysfunction and brain atrophy [1]. Despite differing in affected brain regions and causes, they share common cellular and molecular mechanisms, wherein a gradual loss of endogenous antioxidant buffering capacity is thought to play a major role.

The central nervous system (CNS), comprising the brain and spinal cord, contains neurons, astrocytes, oligodendrocytes, and resident immune cells, with microglia representing the primary phagocytes and sole resident macrophages [2]. Microglia maintain CNS homeostasis by monitoring the microenvironment, clearing debris, phagocytosing pathogens and damaged cells, participating in inflammation, supporting neurons, pruning synapses, and regulating myelin integrity [3,4]. Dysfunctional microglia contribute to aging, neurodegeneration, and disrupted CNS homeostasis, making their preservation a potential therapeutic target [5]. Aging reduces microglial number and activity, alters their morphology, and impairs function, all of which is driven by factors such as telomere shortening, DNA damage, and oxidative stress [3,6].

Oxidative stress is closely linked to aging and age-related diseases, impairing microglial function and triggering neuroinflammatory responses in the brain [7]. Beyond CNS redox imbalance, converging evidence supports the presence of peripheral oxidative vulnerability in AD and its prodromal stages (i.e., mild cognitive impairment or MCI), suggesting that both central and systemic redox dysregulation may contribute to disease biology. Consequently, ROS clearance is considered a critical strategy for the prevention and treatment of neurodegenerative diseases [8,9].

The endogenous antioxidant response protects cells from oxidative stress by upregulating cytoprotective enzymes, which is primarily regulated by the transcription factor Nrf2 [10]. Under normal conditions, Nrf2 is bound to the protein Kelch-like ECH-associated protein 1 (Keap1) in cytosol and is targeted for degradation. Oxidative stress or electrophiles trigger Nrf2 release, allowing its nuclear translocation, heterodimerization with small Maf proteins, and binding to antioxidant response elements (AREs) to activate genes, such as catalase (CAT), superoxide dismutase (SOD), glutathione peroxidase (GPx), NAD(P)H-quinone oxidoreductase 1 (NQO1), and heme oxygenase-1 (HO-1) [11,12,13,14]. Dysregulation of Nrf2 has been increasingly linked to aging and neurodegenerative diseases [12].

Heme oxygenase-1 (HO-1) is a rate-limiting enzyme in heme degradation, producing carbon monoxide, free iron, and biliverdin, which is further converted to bilirubin. These byproducts confer cytoprotective effects, including antioxidants, anti-inflammatory, and anti-apoptotic actions. Beyond its canonical function, HO-1 also has a non-canonical role in cellular signaling, with nuclear localization linked to pathologies such as cancer and neurodegenerative diseases, though its precise nuclear function remains unclear [15]. Oxidative stress can activate this Nrf2/HO-1 pathway, highlighting the need to explore these mechanisms in neurodegeneration and identify molecules that counteract ROS-induced damage. In vitro models often use cell lines and H_2_O_2_-induced oxidative stress to evaluate the protective effects of various compounds [16].

Natural products have long been a key source of new drugs [17,18]. While most research focuses on plants, microorganisms, particularly those from extreme environments like the Antarctic, present a highly diverse and largely untapped resource with significant biotechnological potential [19,20]. Antarctic fungi, exposed to harsh conditions such as low temperatures, freeze–thaw cycles, scarce water, strong winds, and high UV radiation, are especially notable for their diversity and resilience [21]. Among them, *Penicillium* species are widely distributed, economically impactful, and known for producing mycotoxins, such as patulin and ochratoxin A, and lesser-studied secondary metabolites that may hold therapeutic potential [22,23].

In this study, we set out to isolate and identify a molecule from a fungus of the genus *Penicillium*, isolated from soil samples from Deception Island in Antarctica, and test its neuroprotective capacity against oxidative stress caused by hydrogen peroxide (H_2_O_2_), which was evaluated in HMC-3 microglia cells and human peripheral blood mononuclear cells (PBMCs) collected from cognitively healthy older adults and older adults with mild cognitive impairment.

## 2. Materials and Methods

### 2.1. Isolation and Identification of Penicillium sp.

Viridicatin was isolated from the cultures of *Penicillium* sp. isolated from soil samples collected in Antarctica during ECA-57 in 2019 (ECA: Antarctic Scientific Expedition, Project INACH RT-33-18); 2 g of soil was collected from Deception Island (Coordinates: S 62°58′28″ W 60°34′14″), and the sample was transported to University of La Frontera in Temuco, where the fungi was cultured and purified. A 1 g aliquot of the sediment was suspended in 9 mL of sterile saline (0.85% NaCl), vortexed, and serially diluted tenfold and hundredfold in the same saline. From each dilution, 100 µL was plated in triplicate onto YM agar (yeast extract 0.3%, malt extract 0.3%, peptone 0.5%, glucose 2%, agar 2%; pH 6.2 ± 0.2) and supplemented with chloramphenicol at 100 µg mL^−1^ to suppress bacterial growth. Plates were held at 15 °C for 30 days, after which individual colonies were picked and subcultured until pure strains were obtained.

The characterization of the fungi was done by PCR. Genomic DNA was recovered from fresh mycelium with the E.Z.N.A. Fungal DNA Mini Kit (Omega Bio-tek, Norcross, GA, USA), as directed by the supplier. The internal transcribed spacer (ITS) region was amplified using the primer pair ITS1F [24] and ITS4 [25]. Amplicons were sequenced at the AUSTRAL-omics Core Facility (Faculty of Science, Universidad Austral de Chile; https://australomics.cl/, accessed on 5 June 2025), and the forward and reverse chromatograms were assembled and curated in Geneious Prime 2024, Biomatters Ltd. (Auckland, New Zealand). The resulting ITS consensus sequence was queried against the GenBank database using BLAST, https://blast.ncbi.nlm.nih.gov/Blast.cgi (accessed on 5 June 2025) [26].

### 2.2. Extraction and Purification of Viridicatin

For metabolite production, the strain was grown statically in the dark at 15 °C for 23 days across eighty 1 L conical flasks, each containing 400 mL of YM broth together with 10 g of cotton as a support. The mycelium and broth were then separated by filtration, and each fraction was extracted independently with ethyl acetate (EtOAc; three successive extractions). The combined organic phases were concentrated under reduced pressure to give 4.2 g of total organic extract, which was fractionated on a silica gel column eluted with a hexane–EtOAc gradient of increasing polarity. The hexane:EtOAc 1:4 (*v*/*v*) fraction yielded a single solid that, upon recrystallization from EtOAc at 4 °C, produced colorless single crystals.

### 2.3. Structure Elucidation of Viridicatin

The structure of viridicatin was determined by single-crystal X-ray diffraction and 1D and 2D NMR analyses. 1D and 2D-NMR spectra were acquired in deuterated acetone (Sigma-Aldrich, St. Louis, MO, USA) on a Bruker Avance NEO 500 spectrometer (Bruker Biospin GmbH, Rheinstetten, Germany), operating at 500 MHz for ^1^H and 125 MHz for ^13^C. Resonances were assigned from the combined H,H-COSY, NOESY, HSQC, and HMBC correlations.

The crystal structure was determined by single-crystal structure analysis. A single crystal of suitable quality was chosen under a Leica M205C light microscope and mounted in oil. Diffraction data were collected on a Stadivari diffractometer [27], using monochromated Mo-Kα radiation (λ = 0.71073 Å) and corrected with the X-Area v1.75 (STOE and Cie GmbH, Darmstadt, Germany). The structure was solved by direct methods and refined on F^2^ against all reflections by full-matrix least-squares within the SHELX program suite [28,29], applying anisotropic displacement parameters to all non-hydrogen atoms and placing hydrogen atoms at calculated positions. Table S1 was compiled in FinalCif (https://dkratzert.de/finalcif.html, accessed on 5 June 2025), and the structure was rendered in Mercury [30]. The corresponding data (F9ACR; CCDC 2524160) are freely available from the Cambridge Crystallographic Data Centre at http://www.ccdc.cam.ac.uk (accessed on 5 June 2025).

### 2.4. Cell Culture

Human immortalized microglial HMC-3 cells were purchased on December 2022 (ATCC^®^ CRL-3304™, USA). After 6–7 passages, cells were tested for the absence of mycoplasma before and after a routine decontamination procedure. Cell cultures were treated with Plasmocin^®^ Treatment (InvivoGen, cat. ant-mpt) at 25 µg/mL for 14 days following the manufacturer’s recommendations. All the experiments were performed on tested decontaminated cells ranging between 7 and 10 passages (Supplementary Figure S10). Cells were maintained in DMEM containing 10% fetal bovine serum (FBS; Cytiva, Marlborough, MA, USA) with penicillin (100 U/mL) and streptomycin (0.1 mg/mL) (Cytiva, Marlborough, MA, USA) and kept at 37 °C under a humidified 5% CO_2_ atmosphere. Oxidative stress conditions were optimized by exposing cells to increasing concentrations of H_2_O_2_ for 24 h, selecting 300 µM for subsequent experiments. Hydrogen peroxide (H_2_O_2_; 3% solution, suitable for microbiology; Sigma-Aldrich, Product No. 88597) was used as the oxidative stress inducer.

### 2.5. Cell Viability by IncuCyte Assay

Real-time cytoprotection by viridicatin was conducted in HMC-3 microglia seeded in 96-well plates at 5 × 10^3^ cells/well and left for 24 h to reach roughly 80% confluence. The treatments performed were: control cells (no treatment), cells treated with H_2_O_2_ (300 µM) for 24 h; cells treated with viridicatin (25, 50, 100, and 200 µM) for 24 h; and cells pre-incubated with viridicatin (100 µM) for 2 h, after which the culture medium was removed, and then treated with H_2_O_2_ (300 µM) for 24 h. The cell death and cell proliferation parameters were measured. Membrane integrity was tracked on an IncuCyte^®^ S3 live-cell imaging platform (Bohemia, NY, USA) using the cell-impermeant probe Sytox Green (30 nM; Invitrogen™, #S7020, Carlsbad, CA, USA), whose fluorescence marks cells that have lost membrane integrity. The green signal was captured and quantified automatically at hourly intervals over the 24 h run, and the readouts were analyzed in GraphPad Prism 8.0. Culture confluence, taken as the proportion of the image field covered by adherent cells, was measured in parallel using IncuCyte^®^ v2019B software at the Advanced Microscopy Center (CMA Biobío, University of Concepción, Concepción, Chile).

### 2.6. Mitochondrial Membrane Potential Assay

Shifts in mitochondrial membrane potential (ΔΨm) were assessed using the Invitrogen™ JC-1 dye according to the manufacturer’s directions. For this, HMC-3 microglia cells were seeded in a 96-well plate (5000 cells/well), and the different treatments were added: control cells (no treatment), cells with H_2_O_2_ (300 µM), cells pre-incubated with viridicatin (100 µM) for 2 h and then treated with H_2_O_2_ (300 µM) for 24 h (with the culture media was removed). This was done for two different peroxide times: 3 and 6 h.

Once the treatments were finished, the wells were washed with PBS, and the JC-1 probe (1 µM) was added. The fluorescence of both channels was read by the IncuCyte system. The data obtained were processed using GraphPad Prism 8.0 software.

### 2.7. Immunofluorescence and Confocal Microscopy Assay

Confocal immunofluorescence microscopy was performed in HMC-3 cells using primary antibodies against Nrf2, HO-1, and NQO1. Cells were seeded on glass coverslips in 12-well plates (60,000 cells/well) and allowed to adhere for 12 h. Cells were then pre-treated with viridicatin (100 µM) for 2 h, followed by exposure to H_2_O_2_ (300 µM) for 6 h. Experimental conditions included untreated controls, H_2_O_2_-treated cells, viridicatin-treated cells, and co-treated cells with viridicatin and H_2_O_2_ at both time points.

At the end of the treatments, cells were fixed with 4% paraformaldehyde in phosphate-buffered saline (PBS) for 30 min, washed with Tris-HCl buffer (pH 7.8), and then incubated in the same buffer containing 1% bovine serum albumin (BSA) and 0.2% Triton X-100 for 5 min at room temperature. Then, the samples were incubated with the following primary antibodies overnight at room temperature Nrf2 Rabbit mAb #12721 (Cell Signaling), HO-1 Mouse (A-3): sc-136960 (Santa Cruz Biotechnology, INC), and *NQO1* Mouse (A180): sc-32793 (Santa Cruz Biotechnology, INC) at manufacturer-recommended dilutions in a humidified chamber for ~12 h. After three additional BSA washes, cells were incubated with secondary antibodies (Anti-Mouse IgG, #715-035-150; Anti-Rabbit IgG 711-035-152, 1:10,000, Jackson Immunoresearch, USA) for 2 h in the dark. Coverslips were washed with distilled water, mounted on slides, and kept in darkness for 3 h prior to imaging. Confocal images were acquired using immersion oil, and nucleus were counterstained with Hoechst (blue). The slides were analyzed using confocal laser microscopy (Confocal NLO 780, Carl Zeiss, Oberkochen, Germany) at the CMA BIO-BIO (Concepcion University). Data analysis was completed with GraphPad Prism 8.0. Fluorescence intensity was quantified using ImageJ by defining nuclear and cytoplasmic regions of interest (ROIs), and data were expressed as nuclear/cytoplasmic fluorescence ratios.

### 2.8. Protein Analysis by Western Blot

Protein expression was determined by Western blot analysis. HMC-3 cells (4 × 10^5^ cells/well) seeded in P60 plates were incubated in media for 12 h. Then, cells were exposed to viridicatin 100 µM for 3 h before these were stimulated with H_2_O_2_ 300 µM for 6 h. Cells were then rinsed twice with ice-cold PBS and lysed with RIPA (Lysis Buffer System), which was supplemented with 200 mM PMSF (Protease Inhibitor cocktail) and sodium ortho vanadate 100 mM (ChemCruz). Lysates were cleared by centrifugation at 12,000 rpm for 20 min, and the supernatants were quantified using the PierceTM BCA Protein Assay Kit. Equal amounts of protein (30 µg) were heated at 95 °C for 5 min and separated using 10% SDS PAGEM. Following electrophoresis, the proteins were transferred onto a polyvinylidene fluoride membrane (PVDF, GVS Filter Technology, Zola Predosa, Italy). The membranes were blocked with 5% non-fat milk at room temperature for 1 h and then incubated overnight at 4 °C with primary antibodies against Nrf2 Rabbit mAb #12721 (Cell Signaling), HO-1 Mouse (A-3): sc-136960 (Santa Cruz Biotechnology, INC), and NQO1 Mouse (A180): sc-32793 (Santa Cruz Biotechnology, Inc., Dallas, TX, USA). Membranes were then incubated with HRP-conjugated secondary antibodies (1:10,000; Jackson ImmunoResearch, West Grove, PA, USA). Protein bands were visualized using the SuperSignal™ West Pico PLUS Chemiluminescent Substrate (Thermo ScientificTM) in a photodocumenter (G: BOX Chemi XRQ gel doc system, SYNGENE) and band densitometry analysis were performed with Image J software (version 1.54r, National Institutes of Health, Bethesda, MD, USA). αTubulin #2144 (Cell Signaling) was used as an internal control.

### 2.9. Quantitative Real-Time PCR Analysis

HMC-3 cells were cultured and exposed to the following experimental conditions: untreated control cells, cells treated with H_2_O_2_ (300 µM), cells treated with viridicatin (100 µM), and cells pre-treated with viridicatin (100 µM) for 2 h before exposure to H_2_O_2_ (300 µM). Total RNA was isolated using RNA-Solv Reagent (Omega Bio-tek, Inc., Norcross, GA, USA), according to the manufacturer’s instructions. Subsequently, 1 µg of total RNA was reverse-transcribed into cDNA using the High-Capacity cDNA Reverse Transcription Kit (Applied Biosystems, Foster City, CA, USA) following the manufacturer’s protocol. The resulting cDNA was used to evaluate the mRNA expression levels of *NFE2L2* (NM_006164.5), *NQO1* (NM_000903.3), and *HMOX1* (NM_002133.3) by quantitative real-time PCR. *RPL27* (NM_000988.5) was used as the internal reference gene for normalization. qPCR reactions were performed using HOT FIREPol^®^ EvaGreen^®^ qPCR Mix Plus (Solis BioDyne OÜ, Tartu, Estonia) on a Bioneer Exicycler™ 96 Real-Time Quantitative PCR System (Bioneer Corporation, Daejeon, Republic of Korea). Reactions were run for 40 amplification cycles.

The primer sequences used were as follows: *NFE2L2*/Nrf2, forward 5′-TGCCCCTGGAAGTGTCAAAC-3′ and reverse 5′-CCCCTGAGATGGTGACAAGG-3′; NQO1, forward 5′-GGATTGGACCGAGCTGGAAA-3′ and reverse 5′-CAGCCGTCAGCTATTGTGGA-3′; *HMOX1*/HO-1, forward 5′-AGTCTTCGCCCCTGTCTACT-3′ and reverse 5′-CTGGTGTGTAGGGGATGACC-3′; and *RPL27*, forward 5′-TCCGGACGCAAAGCTGTCATC-3′ and reverse 5′-GGTCAATTCCAGCCACCAGAGCAT-3′. Specific primers were obtained from Integrated DNA Technologies (Coralville, IA, USA). Relative gene expression was calculated using the 2^−ΔΔCt^ method, with *RPL27* as the internal reference gene and untreated control cells as the calibrator condition.

### 2.10. Human Participants and Ethical Approval

Older adults aged 60 years and older were recruited and classified as either cognitively healthy (CH, *n* = 4) or as having mild cognitive impairment (MCI/mild AD, *n* = 8) based on standardized clinical and neuropsychological assessments, following the National Institute on Aging–Alzheimer’s Association [31] diagnostic criteria. Cognitive status was evaluated using a battery of validated instruments, including the Montreal Cognitive Assessment (MoCA), the MoCA—Memory Index Score (MoCA-MIS), AD8, and the Clinical Dementia Rating—Sum of Boxes (CDR-SB). The study protocol was approved by the ethics committee of Hospital Clínico de la Universidad de Chile (Acta N° 035, approval date 5 June 2019) and conducted in accordance with the Declaration of Helsinki. Given the participants’ diagnosis of mild cognitive impairment (MCI), their decisional capacity was individually assessed prior to inclusion. While participants provided written informed consent, in cases where limited decisional capacity was determined, consent for the collection of samples was provided by their legally authorized representatives (LARs) or next of kin, with the patients’ assent obtained whenever possible.

### 2.11. PBMC Isolation and Oxidative Stress-Induced Viability Assay

Peripheral blood mononuclear cells (PBMCs) were isolated from venous blood samples obtained from cognitively healthy (CH) and mild cognitive impairment (MCI) donors using SepMate™ tubes according to the manufacturer’s instructions. Cell counts and viability were determined using acridine orange staining and a LUNA-FL automated cell counter. PBMCs were seeded at a density of 90,000 cells per well in 96-well plates and maintained in RPMI 1640 medium supplemented with 10% fetal bovine serum (FBS). Cells were pre-treated for 2 h with viridicatin dissolved in supplemented RPMI medium (final concentrations: 100 µM for PBMCs from MCI donors and 50 µM for PBMCs from CH donors), or with the vehicle control (RPMI medium containing DMSO).

Following pre-treatment, PBMCs were exposed to hydrogen peroxide (H_2_O_2_) to induce acute oxidative stress. PBMCs from MCI donors were treated with 100 µM or 300 µM H_2_O_2_, whereas PBMCs from CH donors were treated with 300 µM H_2_O_2_. Cells were incubated for an additional 20 h at 37 °C in a humidified atmosphere containing 5% CO_2_. Untreated cells (PBS only) were used to define basal viability, and methanol-treated cells were included as a positive control for cell death. Cell viability was assessed by flow cytometry using propidium iodide (PI) exclusion. After treatment, cells were collected, resuspended in 200 µL of PI solution (1:100 in PBS), and analyzed on a FACS Canto II cytometer. For each donor, paired measurements were obtained for vehicle- and viridicatin-treated conditions under identical oxidative stress levels. Viability data were expressed as percentage relative to untreated cells from the same donor, which were defined as 100%. Each data point represents an independent donor from four to eight patients per condition and averaged from two technical replicates.

Normality of the data was assessed using the Shapiro–Wilk test. Paired comparisons between vehicle- and viridicatin-treated conditions under the same H_2_O_2_ concentration were performed using paired *t*-tests for normally distributed data or Wilcoxon signed-rank tests for non-normally distributed data. Statistical analyses were performed using GraphPad Prism version 8.

### 2.12. Molecular Docking

Molecular docking studies were performed to evaluate the potential binding interactions between viridicatin and key proteins of the Nrf2/HO-1 antioxidant pathway using PyRx 0.8 with integrated AutoDock Vina 1.1.2 following protocols used by our lab [32]. Three target proteins were selected based on our in vitro findings: KEAP1 Kelch domain (PDB: 4IQK), HO-1 (PDB: 3CZY), and *NQO1* (PDB: 1D4A). The three-dimensional crystal structures were retrieved from the RCSB Protein Data Bank (https://www.rcsb.org). Protein preparation involved the removal of water molecules, co-crystallized ligands, and non-essential chains, followed by the addition of polar hydrogens and Gasteiger charges using AutoDock Tools. The three-dimensional structure of viridicatin was generated using ChemDraw 15.0 and optimized by energy minimization prior to docking.

The docking grid box was defined to encompass the active site residues of each target protein. For KEAP1 (4IQK), the grid was centered on the Nrf2-binding pocket of the Kelch domain with dimensions of 25 × 25 × 25 Å, covering the key residues involved in the KEAP1-Nrf2 protein–protein interaction including Arg380, Asn382, Arg415, Arg483, Ser508, and Tyr525. For HO-1 (3CZY), the grid was positioned around the heme-binding cavity with dimensions of 22 × 22 × 22 Å, encompassing the catalytic site including His25, Thr26, and the heme cofactor. For *NQO1* (1D4A), the grid was centered on the FAD-binding domain with dimensions of 20 × 20 × 20 Å, covering the active site residues including Tyr128, Phe178, and the FAD-cofactor-binding region. The exhaustiveness parameter was set to 8 for all docking runs.

Docking results were evaluated based on binding affinity (kcal/mol), and the best pose for each target was selected according to the lowest binding energy. Protein–ligand interactions including hydrogen bonds, hydrophobic contacts, and π-interactions were analyzed and visualized using BIOVIA Discovery Studio Visualizer, Dassault Systèmes (San Diego, CA, USA) [33]. Two-dimensional and three-dimensional interaction diagrams were generated to illustrate the binding mode and key molecular contacts.

### 2.13. Molecular Dynamics Simulations and MM-GBSA Calculations

Molecular dynamics simulations were performed for the three viridicatin–protein complexes (KEAP1, PDB: 4IQK; HO-1, PDB: 3CZY; and NQO1, PDB: 1D4A) using the Desmond module (Schrödinger v2019-4). The best docking poses were used as starting configurations. Each system was solved in a TIP3P orthorhombic water box with a 10 Å buffer, neutralized with counterions, and an ionic strength set to 0.15 M NaCl. Simulations were run for 100 ns under the NPT ensemble (300 K, 1.013 bar) using the OPLS3e force field. Trajectory analysis (RMSD, RMSF, protein–ligand contacts) was performed using the built-in Desmond tools. Dynamic cross-correlation matrices (DCCMs) and principal component analysis (PCA) are reported in the Supplementary Materials. Endpoint MM-GBSA binding free energy calculations were performed using the Prime module on frames extracted at 0 ns and 100 ns from each trajectory.

### 2.14. ADMET and Drug-Likeness Prediction

The drug-likeness and pharmacokinetic profile of viridicatin were evaluated using SwissADME (http://www.swissadme.ch) and ADMETlab 3.0 (https://admetmesh.scbdd.com) [34]. Toxicity endpoints were predicted using ProTox-3.0 (https://tox.charite.de/protox3). The canonical SMILES notation of viridicatin (Oc1c(=O) [nH]c2c(c1c1ccccc1) cccc2) was used as input for all computational predictions.

## 3. Results

### 3.1. Isolation and Structural Elucidation of Viridicatin from Penicillium sp.

**Figure 1:**
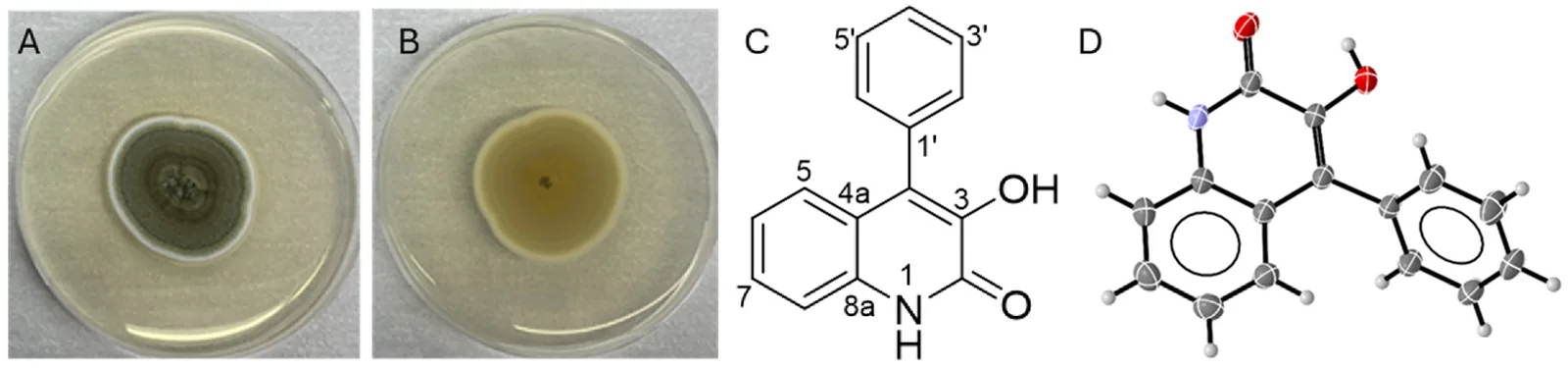
(**A**,**B**) Representative colony morphology of *Penicillium* sp. grown on YM agar for 21 days, showing the obverse (**A**) and reverse (**B**) sides. (**C**) Molecular structure of viridicatin. (**D**) ORTEP representation of viridicatin determined by single-crystal X-ray diffraction, with displacement ellipsoids drawn at the 50% probability level. In (**D**), Carbon atoms are shown in grey, nitrogen in blue, oxygen in red, and hydrogen in white.

Viridicatin is an alkaloid previously isolated from different fungal strains mainly belonging to the genus *Penicillium*, and its crystal structure has previously been reported in the literature [35]. Now, we isolated viridicatin as the major secondary metabolite from static cultures of *Penicillium* sp. obtained from Antarctic soil samples collected from Deception Island. After extraction and chromatographic purification, a single pure compound was obtained in the form of colorless crystals. The molecular structure of viridicatin was unambiguously established by combined spectroscopic and crystallographic analyses. Single-crystal X-ray diffraction analysis confirmed the molecular structure and provided definitive stereochemical and conformational information (Figure 1C,D). Crystallographic parameters are reported in Table S1, and the structural data have been deposited in the Cambridge Crystallographic Data Centre (CCDC 2524160). To our knowledge, this represents the first crystallographic structure reported for viridicatin, providing unequivocal structural confirmation of this Antarctic-derived fungal metabolite. The ^1^H and ^13^C NMR spectra, together with HSQC, are key long-range HMBC correlations supporting the quinolinone scaffold and are summarized in the Supplementary Materials (Table S2 and Figures S2–S9).

### 3.2. H_2_O_2_-Induced Cytotoxicity in HMC-3 Microglial Cells

**Figure 2:**
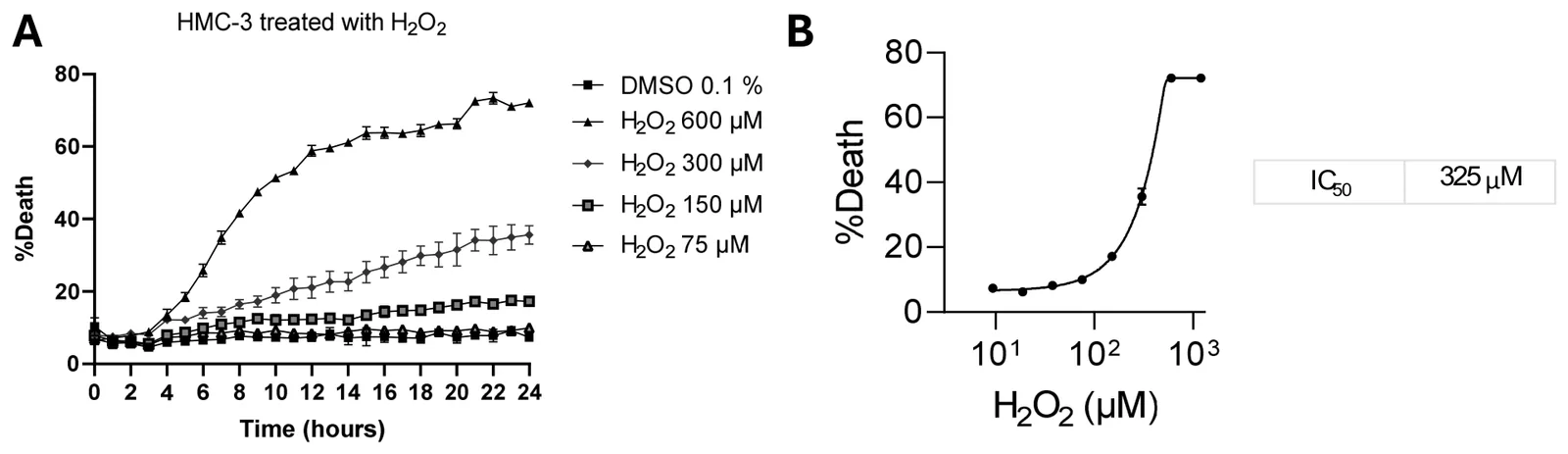
H_2_O_2_-induced cytotoxicity in HMC-3 microglial cells. (**A**) Real-time cell death kinetics measured using the Sytox Green probe in the IncuCyte system for cells treated with H_2_O_2_ between 75 and 600 µM. (**B**) Death curve of H_2_O_2_ and IC_50_ calculation at 24 h (mean ± SD, *n* = 3). The boxed inset in (**B**) indicates the calculated IC50 value. Data are presented as mean ± SD, where SD is the standard deviation and n is the number of independent experiments.

Exposure of HMC-3 microglial cells to increasing concentrations of H_2_O_2_ for 24 h resulted in a marked, concentration-dependent reduction in cell viability becoming more evident from 4 h, as assessed by real-time Incucyte cell culture analysis (Figure 2A). Concentrations ≥ 300 µM caused a pronounced loss of viability, reaching a reduction of approximately 80% compared to untreated controls (Figure 2A). Considering only the end-point measurements (24 h of treatment), a curve was plotted to estimate the IC_50_ of hydrogen peroxide, yielding a value of 325 µM (Figure 2B).

### 3.3. Viridicatin Attenuates H_2_O_2_-Induced Cell Death in HMC-3 Microglial Cells

**Figure 3:**
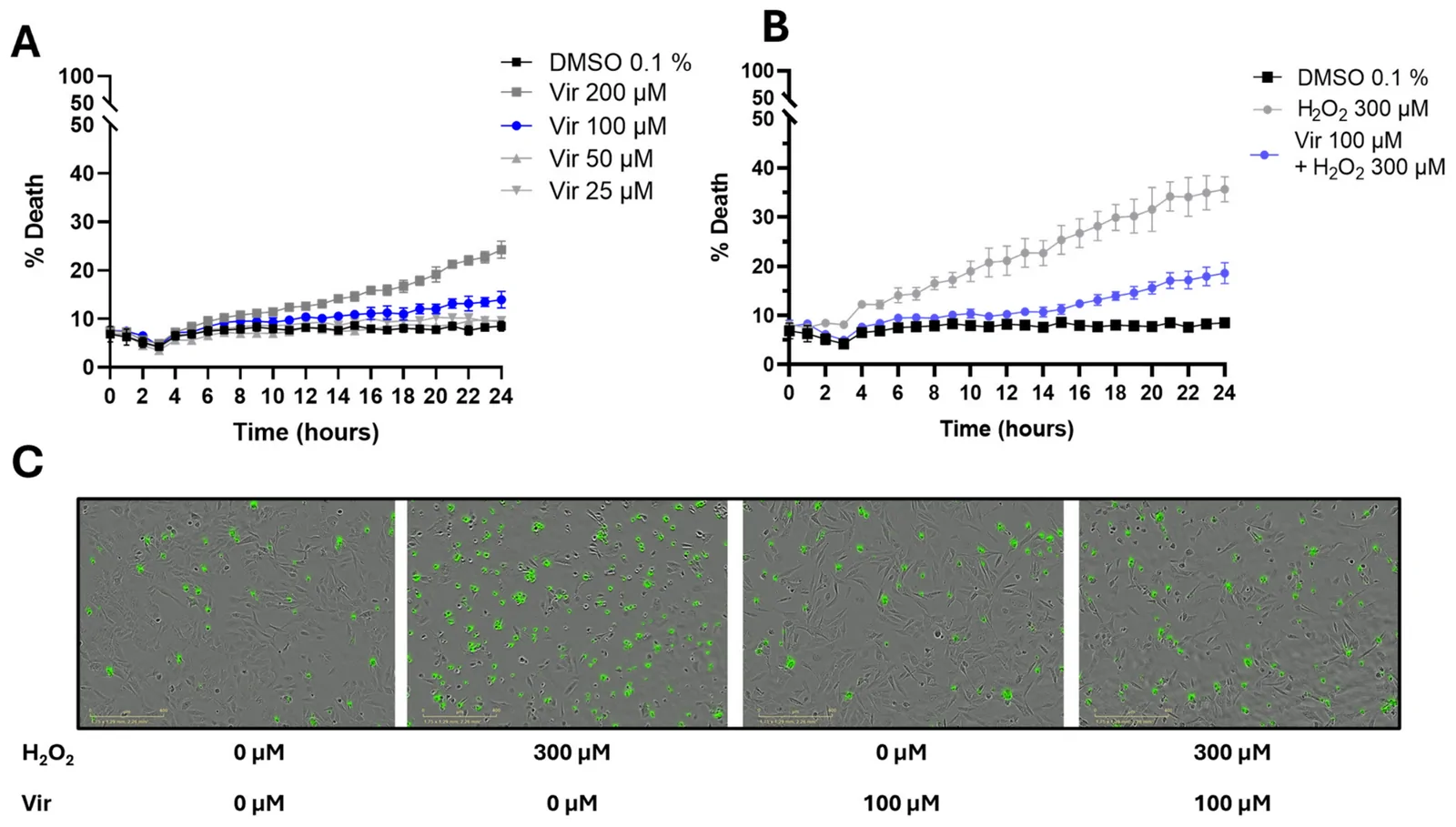
Viridicatin attenuates H_2_O_2_-induced cytotoxicity in HMC-3 microglial cells. (**A**) Sytox-Green-based cell death curves for HMC-3 cells treated with viridicatin. (**B**) Real-time cell death kinetics measured using the IncuCyte system of HMC-3 pre-treated with viridicatin (100 µM, 2 h) and exposed to H_2_O_2_ (300 µM) for 24 h. Data are expressed as percentage of viability relative to untreated controls (mean ± SD, *n* = 3). (**C**) Representative IncuCyte images showing Sytox-Green-positive cells (green) under the indicated conditions after 24 h.

In Figure 3A, it is observed that viridicatin did not cause obvious cell death on its own in the HMC-3 microglia cell line until 100 μM despite a concentration of 200 μM resulting in 20% cell death; therefore, it was decided to use concentrations of viridicatin at 100 μM in further the cell-protection assays.

Pre-treatment of HMC-3 cells with viridicatin 100 µM significantly attenuated H_2_O_2_-induced cytotoxicity (Figure 3B). Sytox Green fluorescence revealed a substantial increase in cell death overtime in H_2_O_2_-treated cultures, rising to 35.6% after 24 h, whereas pre-treatment with viridicatin (100 µM) significantly reduced cell death to 18.6%, as evidenced by a lower accumulation of Sytox-positive cells (green) throughout the 24 h observation period (Figure 3C).

### 3.4. Viridicatin Preserves Mitochondrial Membrane Potential in HMC-3 Microglial Cells Under Oxidative Stress

**Figure 4:**
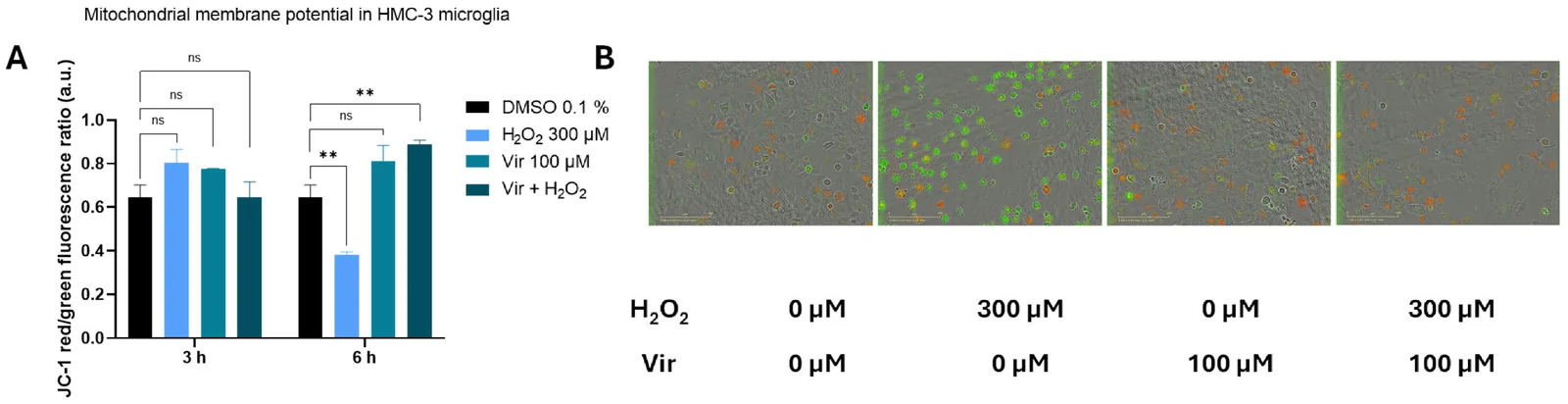
Viridicatin preserves mitochondrial function in HMC-3 microglial cells under oxidative stress. (**A**) Mitochondrial membrane potential (ΔΨm) in HMC-3 cells, assessed by the JC-1 red/green fluorescence ratio after 3 h and 6 h of treatment under the indicated conditions. (**B**) Representative IncuCyte images showing JC-1 staining, where red fluorescence indicates polarized mitochondria and green fluorescence indicate mitochondrial depolarization. Data are expressed as mean ± SD from three independent experiments. Statistical analysis was performed using two-way ANOVA followed by Dunnett’s multiple comparisons test (** *p* < 0.01; ns, not significant).

H_2_O_2_ induced a significant loss of mitochondrial membrane potential compared to the control cells, as assessed by the JC-1 red/green fluorescence ratio (Figure 4A). This activity is evident at 6 h, but not at 3 h for any treatment. At 6 h, microglia cells co-treated with viridicatin (100 µM) and H_2_O_2_ did not lose the mitochondrial membrane potential which maintained higher red/green fluorescence ratios comparable to the control cells. Representative IncuCyte images confirmed these findings, showing predominant green fluorescence in H_2_O_2_-treated cells and preserved red fluorescence in viridicatin-treated and co-treated cells (Figure 4B).

### 3.5. Viridicatin Modulates Nrf2 Translocation to the Nucleus

**Figure 5:**
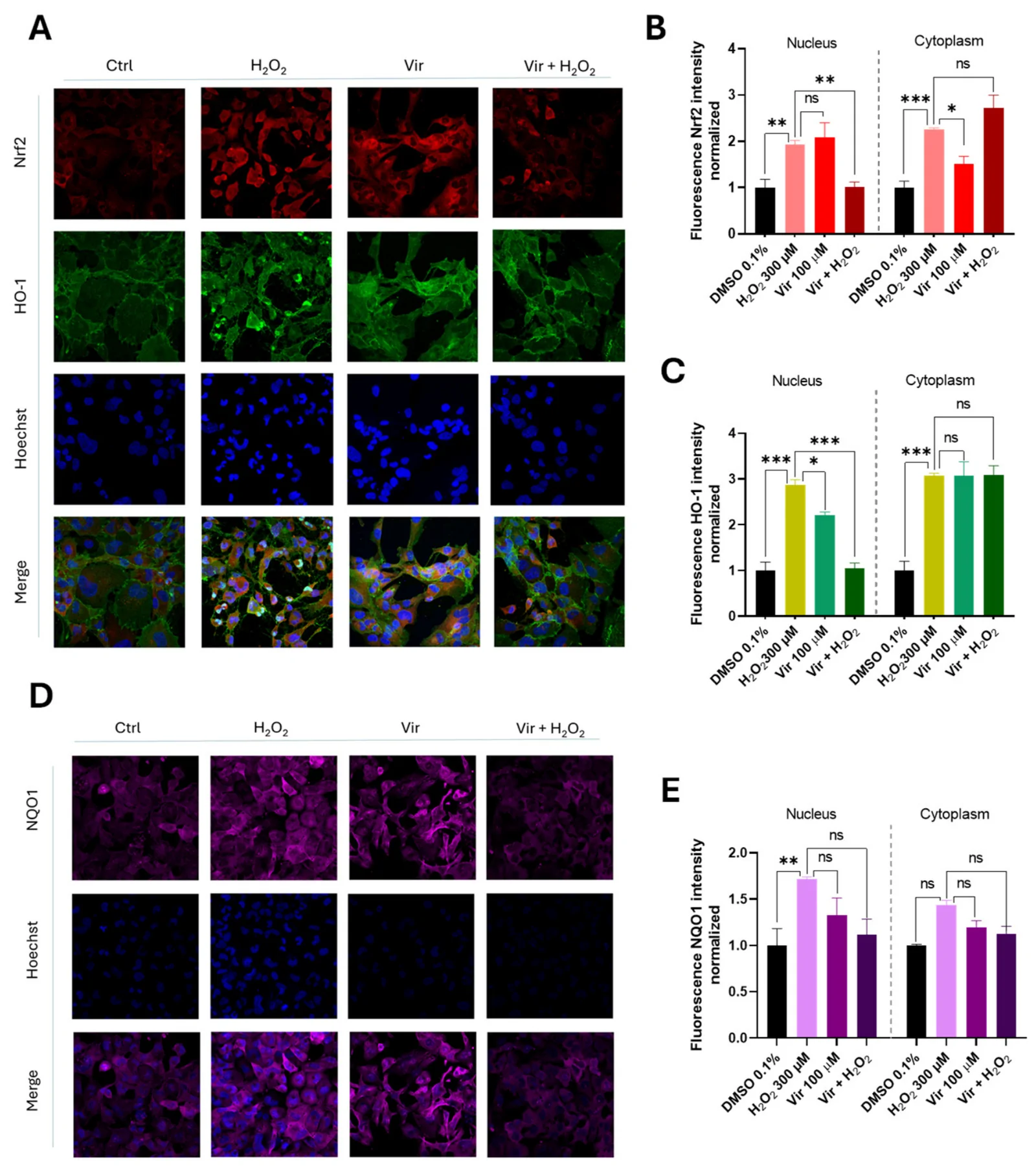
Viridicatin (vir) modulates Nrf2/HO-1/*NQO1* in HMC-3 microglial cells under oxidative stress. (**A**) Representative confocal immunofluorescence images showing Nrf2 (red) and HO-1 (green) localization in HMC-3 cells after 6 h of treatment. Nuclei were counterstained with Hoechst (blue). (**B**) Quantification of Nrf2 fluorescence intensity in nuclear and cytoplasmic compartments (red). (**C**) Quantification of HO-1 fluorescence intensity in nuclear and cytoplasmic compartments (green). (**D**) Representative confocal immunofluorescence images showing *NQO1* localization (magenta) in HMC-3 microglial cells after 6 h of treatment under the indicated conditions. Nuclei were counterstained with Hoechst (blue). Merged images are shown in the bottom panels. (**E**) Quantification of *NQO1* fluorescence intensity in nuclear and cytoplasmic compartments after 6 h of treatment. Cells were treated with vehicle control (DMSO 0.1%), H_2_O_2_ (300 µM), viridicatin (100 µM; 2 h pre-treatment), or viridicatin, followed by treatment with H_2_O_2_. Data are expressed as mean ± SD from three independent experiments. Statistical analysis was performed using two-way ANOVA, followed by Dunnett’s multiple comparisons test (* *p* < 0.05, ** *p* < 0.01, *** *p* < 0.001; ns, not significant).

To determine whether viridicatin modifies the oxidative stress response triggered by H_2_O_2_, the subcellular distributions of Nrf2, HO-1, and *NQO1* were assessed by confocal immunofluorescence at 6 h (Figure 5A).

After treatment, H_2_O_2_ significantly increased Nrf2 abundance, both in the cytoplasm and nucleus, relative to the vehicle-treated cells, indicating activation of the canonical oxidative stress response (Figure 5B). On the other hand, viridicatin also increases the Nrf2 in the nucleus but in a lesser proportion. Importantly, treatment with viridicatin significantly restoring nuclear Nrf2 levels to values comparable to the vehicle control, indicating that viridicatin modulates the H_2_O_2_-driven Nrf2 response.

In addition to Nrf2, the enzymes HO-1 and *NQO1* were evaluated by confocal immunofluorescence. HO-1 and *NQO1* are downstream oxidative stress-responsive proteins of the Nrf2/ARE pathway. H_2_O_2_ treatment increased HO-1 and *NQO1* immunoreactivity relative to the vehicle control, with enhanced staining observed in both nuclear and cytoplasmic compartments (Figure 5C–E). Viridicatin also increased the signal of both enzymes in each compartment.

Importantly, treatment with viridicatin and H_2_O_2_ increased the cytoplasmatic HO-1 signal. Whereas, in the nucleus, HO-1 returned values close to those of vehicle-treated controls. *NQO1* immunoreactivity was weaker than that observed for HO-1 at 6 h. Although some treatment-dependent variations were detected in its subcellular distribution, these changes were less pronounced than those observed for HO-1 and should not be interpreted as an increase in total *NQO1* protein abundance.

### 3.6. Viridicatin Increases Nrf2 and HO-1 in HMC-3 Microglial Cells

**Figure 6:**
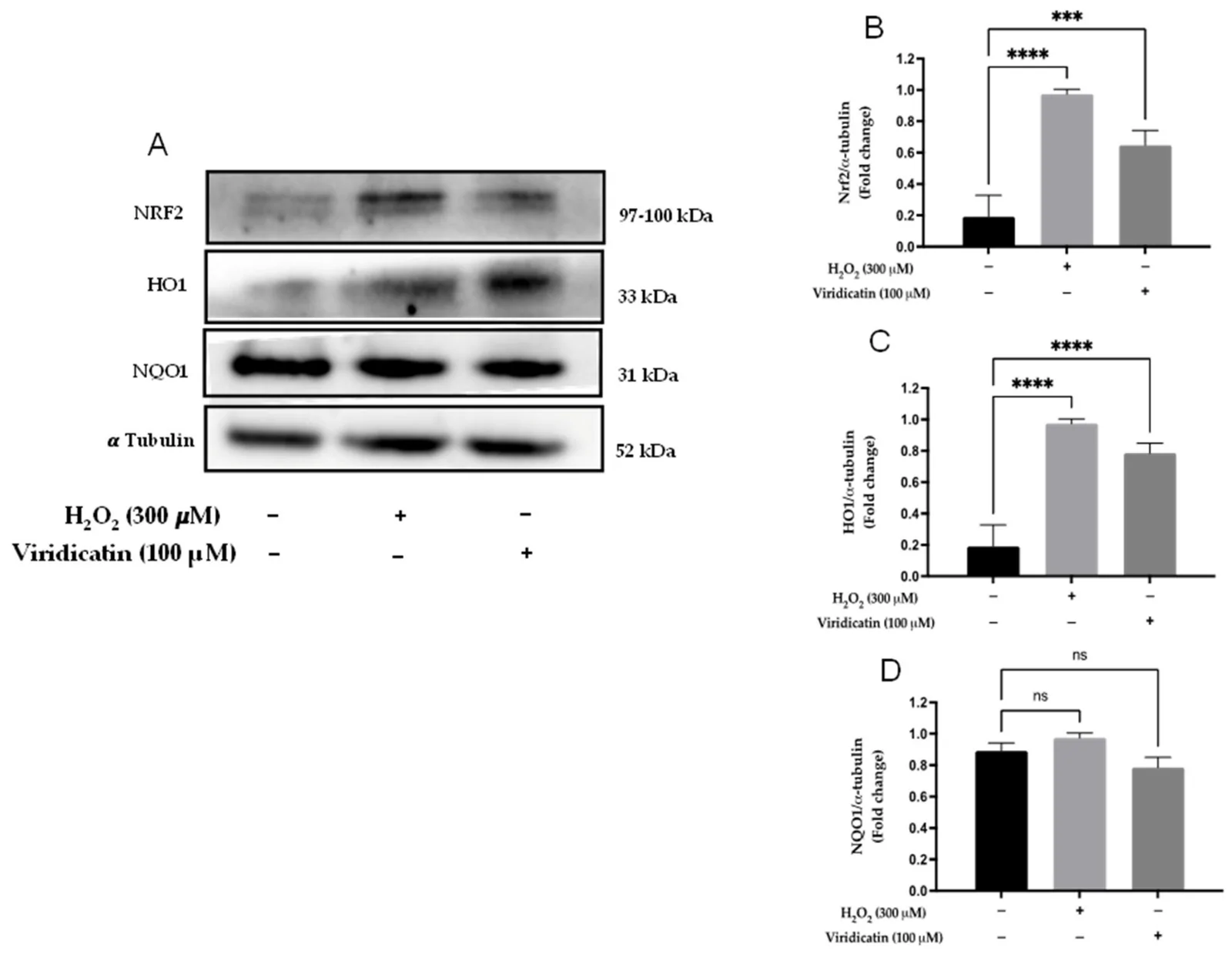
Viridicatin (vir) enhances the Nrf2 pathway. HMC-3 cells were treated with H_2_O_2_ 300 µM or viridicatin for 6 h. (**A**) Representative Western blot images of Nrf2, HO-1, NQO1, and α-tubulin. Protein expression of Nrf2 (**B**), HO-1 (**C**), and *NQO1* (**D**) was detected by Western blot analysis relative to untreated cells. Data are shown as mean ± SD from independent experiments. Statistical analysis was performed using one-way ANOVA, followed by Dunnett’s multiple comparisons test (*** *p* < 0.001, **** *p* < 0.0001; ns, not significant).

The protein expression caused by viridicatin and H_2_O_2_ was evaluated by Western blot analysis. After treatment, viridicatin and H_2_O_2_ significantly increased Nrf2 and HO-1 abundance relative to the vehicle-treated cells (Figure 6A–C). In contrast, *NQO1* protein abundance showed less pronounced variations and was not significantly increased by viridicatin under the conditions evaluated (Figure 6D).

### 3.7. Viridicatin Modulates Antioxidant Enzyme Gene Expression in HMC-3 Microglial Cells Exposed to H_2_O_2_

**Figure 7:**
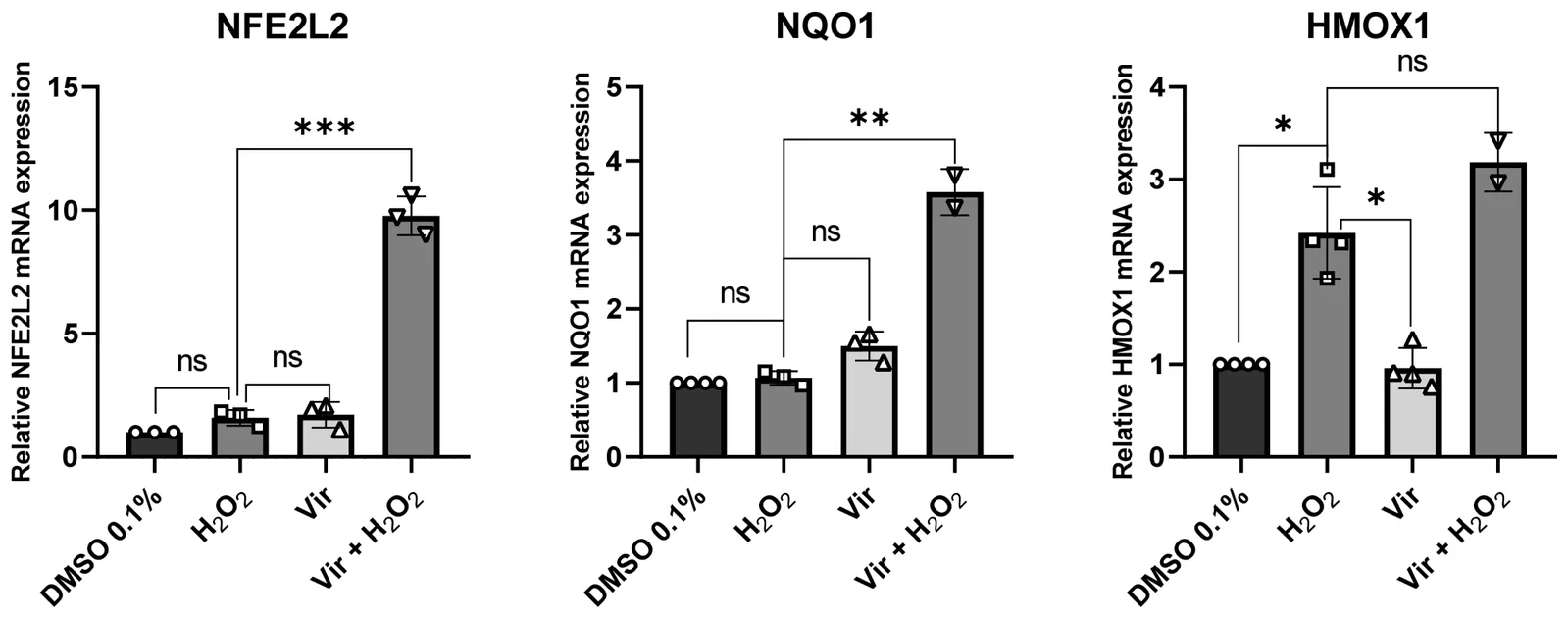
Viridicatin (vir) enhances the antioxidant response under oxidative stress. HMC-3 cells were treated with DMSO 0.1%, H_2_O_2_, viridicatin, or viridicatin plus H_2_O_2_ for 6 h. The relative mRNA expression of *NFE2L2*, NQO1, and *HMOX1* was analyzed by RT-qPCR, normalized to the corresponding endogenous control, and expressed as the fold change relative to DMSO 0.1%. Statistical analysis was performed on ΔCt values using one-way ANOVA followed by planned multiple comparisons. Data are expressed as mean ± SD from three independent experiments. Statistical analysis was performed using one-way ANOVA, followed by Dunnett’s multiple comparisons test. * *p* < 0.05; ** *p* < 0.01; *** *p* < 0.001; ns, not significant.

Quantitative real-time PCR analysis revealed that exposure to H_2_O_2_ induced changes in the expression of *NFE2L2*, NQO1, and *HMOX1* genes (Figure 7). After 6 h of treatment, H_2_O_2_ induced a clear increase in *HMOX1* mRNA expression, with an approximately twofold relative to DMSO 0.1%, consistent with the activation of an oxidative stress response. In contrast, *NQO1* expression was only slightly affected by H_2_O_2_ alone. Under basal conditions, viridicatin did not markedly increase *HMOX1* or *NQO1* expression and produced only modest changes in the antioxidant markers evaluated. Notably, the combination of viridicatin with H_2_O_2_ significantly enhanced *NFE2L2* and *NQO1* mRNA expression compared with H_2_O_2_ alone, reaching the highest fold-change values among the conditions evaluated. These findings indicate that viridicatin potentiates an early transcriptional antioxidant response under oxidative stress conditions, particularly involving *NFE2L2* and NQO1.

### 3.8. Viridicatin Attenuates Oxidative Stress-Induced Loss of Viability in Patient-Derived PBMCs in a Dose- and Cohort-Dependent Manner

**Figure 8:**
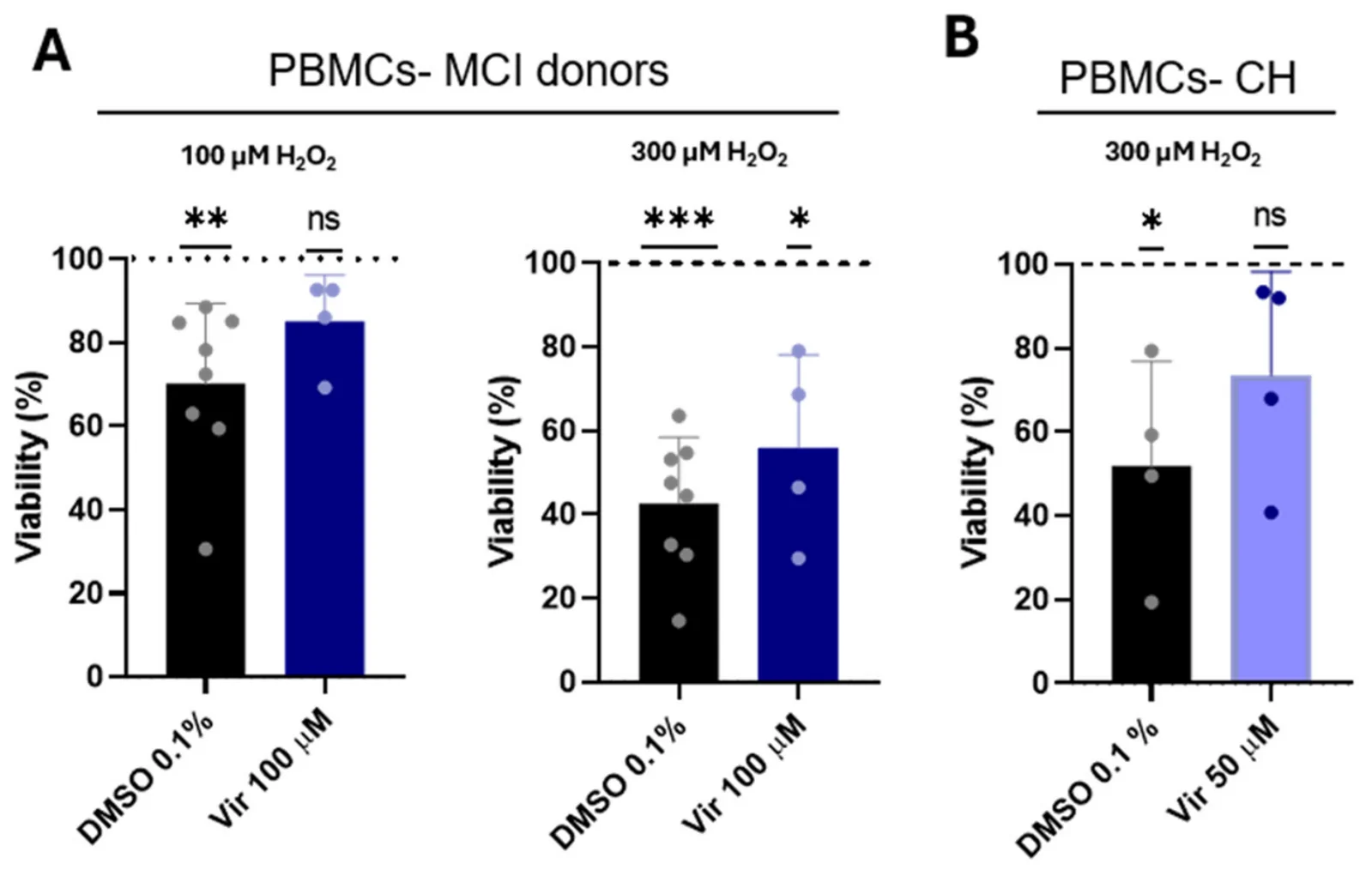
Viridicatin limits oxidative stress-induced loss of viability in PBMCs from older adults. PBMCs obtained from cognitively healthy (CH) and mild cognitive impairment (MCI) donors (>60 years) were exposed to an acute hydrogen peroxide (H_2_O_2_) challenge in the presence of vehicle or viridicatin. Cell viability was assessed by propidium iodide exclusion and expressed as percentage relative to untreated cells (100%, dotted line). (**A**) PBMCs from MCI donors exposed to 100 µM or 300 µM H_2_O_2_ and treated with vehicle or viridicatin (100 µM). (**B**) PBMCs from CH donors exposed to 300 µM H_2_O_2_ and treated with vehicle or viridicatin (50 µM). Bars represent the group mean, and dots represent individual donors. Statistical significance was assessed using one-sample *t* tests comparing each condition against untreated controls (* *p* < 0.05, ** *p* < 0.01, *** *p* < 0.001; ns, not significant).

A body of evidence has supported that the resilience to oxidative stress is altered in mental and neurodegenerative disorders, both systemically and across distinct cell populations. For instance, peripheral blood mononuclear cells (PBMCs) collected from elderly individuals with mild cognitive impairment (MCI) have shown decreased resilience to oxidative challenge when compared to cognitively healthy (CH) age-matched controls. This experimental setting is therefore well-suited to reveal the translational potential of viridicatin. Accordingly, we isolated PBMCs from CH and MCI donors (>60 years) and submitted them to an acute oxidative challenge with hydrogen peroxide (see Materials and Methods) in the absence or presence of viridicatin. In PBMCs from MCI donors, exposure to 100 μM H_2_O_2_ significantly reduced viability to ~70% relative to the untreated condition (defined as 100%). Whereas treatment with viridicatin 100 μM prevented this effect, restricting cell death, and preserving viability at ~85% (Figure 8A). When H_2_O_2_ increased to 300 μM PBMC viability in MCI donors further reduced to ~40% (Figure 8A). Against this condition (300 μM H_2_O_2_), viridicatin 100 μM also protected viability by ~15% (Figure 8A). Therefore, we performed the same experiment but used PBMCs from cognitively healthy donors. In these control patients, the same oxidative challenge (300 μM H_2_O_2_) significantly decreased viability by ~50% relative to untreated controls (Figure 8B). However, even 50 μM of viridicatin (half dose used in MCI patients) significantly abolished cell death induced by H_2_O_2_, preserving viability at ~85% (Figure 8B). The demographic data describing this cohort is included in Table 1. Because interpretation of cytoprotection requires a defined basal reference, we included a supplementary analysis of non-stimulated conditions in CH- and MCI-derived cells. Under basal conditions, untreated cells and their corresponding vehicle- and viridicatin-treated controls displayed similar viability, whereas methanol caused the expected loss of cell survival (Supplementary Figure S11). This indicates that viridicatin neither enhances viability per se nor induces detectable toxicity in the absence of oxidative stress, supporting its specific protective effect during H_2_O_2_ challenge. Together, these results support a protective role of viridicatin in patient-derived PBMCs facing oxidative challenges, with efficacy likely depending on baseline cellular buffering capacity or resilience to oxidative stress (MCI vs. CH).

### 3.9. Molecular Docking of Viridicatin Against the Nrf2 Regulatory Axis

The cellular data indicated that viridicatin acts on the Nrf2 regulatory axis, so molecular docking was used to investigate which protein of this axis engages most favorably with it. Three targets were chosen directly from the experimental observations: the Kelch domain of KEAP1, which controls Nrf2 stability; HO-1, whose nuclear accumulation under oxidative stress was reduced by viridicatin; and NQO1, a downstream Nrf2 target whose levels shifted upon co-treatment.

**Figure 9:**
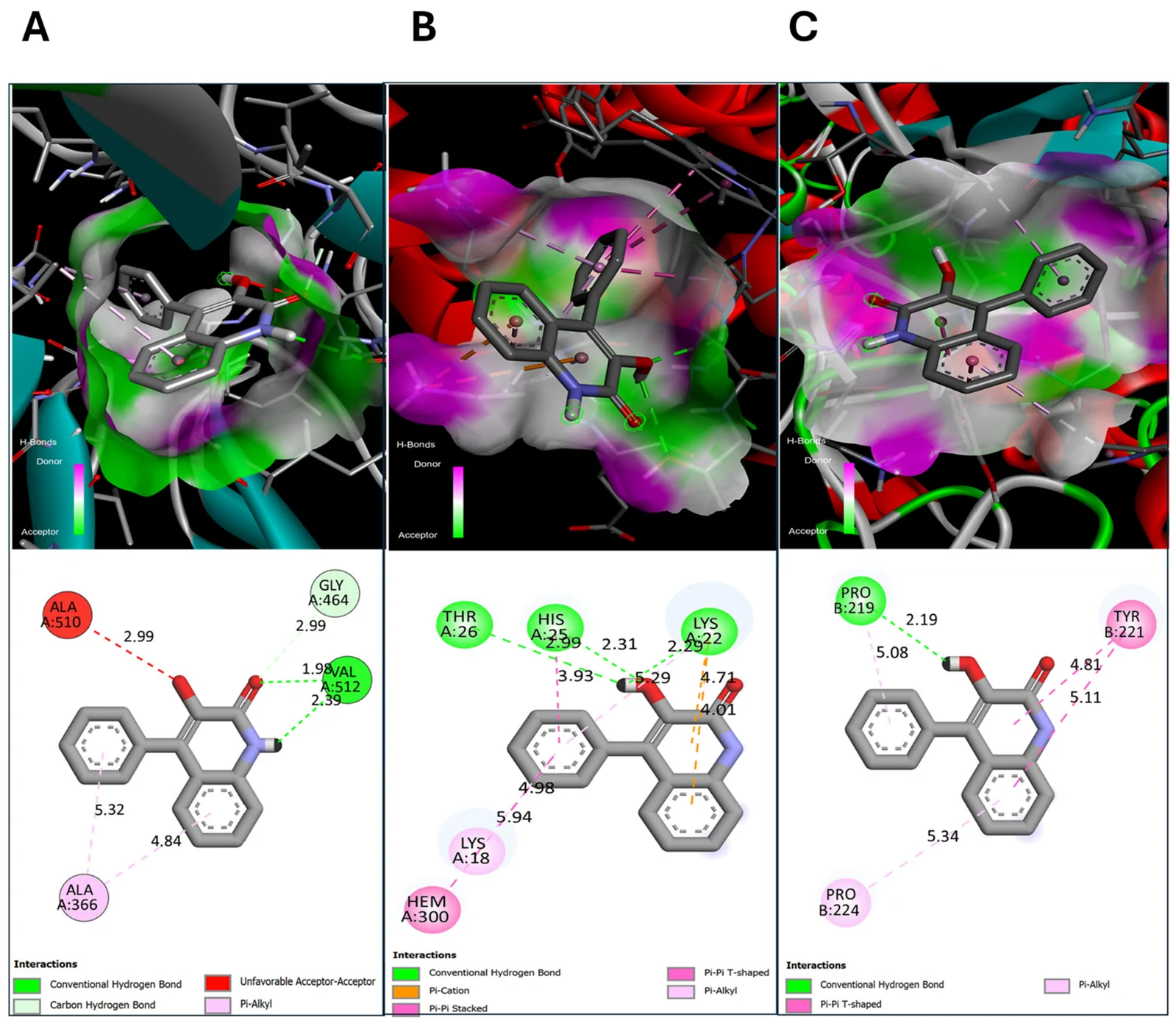
Two-dimensional and three-dimensional molecular docking interaction diagrams of viridicatin with (**A**) KEAP1 Kelch domain (PDB: 4IQK), (**B**) HO-1 (PDB: 3CZY), and (**C**) *NQO1* (PDB: 1D4A). Conventional hydrogen bonds are depicted as green dashed lines; carbon–hydrogen bonds as light green; alkyl and π-alkyl interactions as purple/pink. Distances are indicated in Ångströms (Å).

Viridicatin bound the KEAP1 Kelch domain with the highest predicted affinity (−8.0 kcal/mol; PDB 4IQK), seating within the β-propeller pocket that normally accommodates Nrf2 (Table 2; Figure 9A). The pose was stabilized by one conventional hydrogen bond to Gly464 (2.99 Å), a carbon–hydrogen bond to Val512 (2.79 Å), and alkyl contacts with Ala366 and Ala510. HO-1 ranked second (−7.5 kcal/mol; PDB 3CZY), with viridicatin lodged in the heme cavity through three hydrogen bonds to Thr26, His25, and Lys22 (Figure 9B). *NQO1* gave the weakest affinity (−6.1 kcal/mol; PDB 1D4A), engaging the FAD region through a single hydrogen bond to Pro219 (Figure 9C). The ranking places KEAP1 ahead of HO-1 and well ahead of NQO1, consistent with the interpretation that the *NQO1* changes seen in cells reflect transcription downstream of Nrf2 rather than a direct interaction with the compound.

### 3.10. Molecular Dynamics Simulations of the Viridicatin Complexes

Docking scores a single static pose, so each complex was carried into a 100 ns simulation to test whether the predicted binding survives in explicit solvent. Stability was read from three complementary measures: backbone and ligand RMSD, per residue RMSF, and the persistence of protein–ligand contacts.

**Figure 10:**
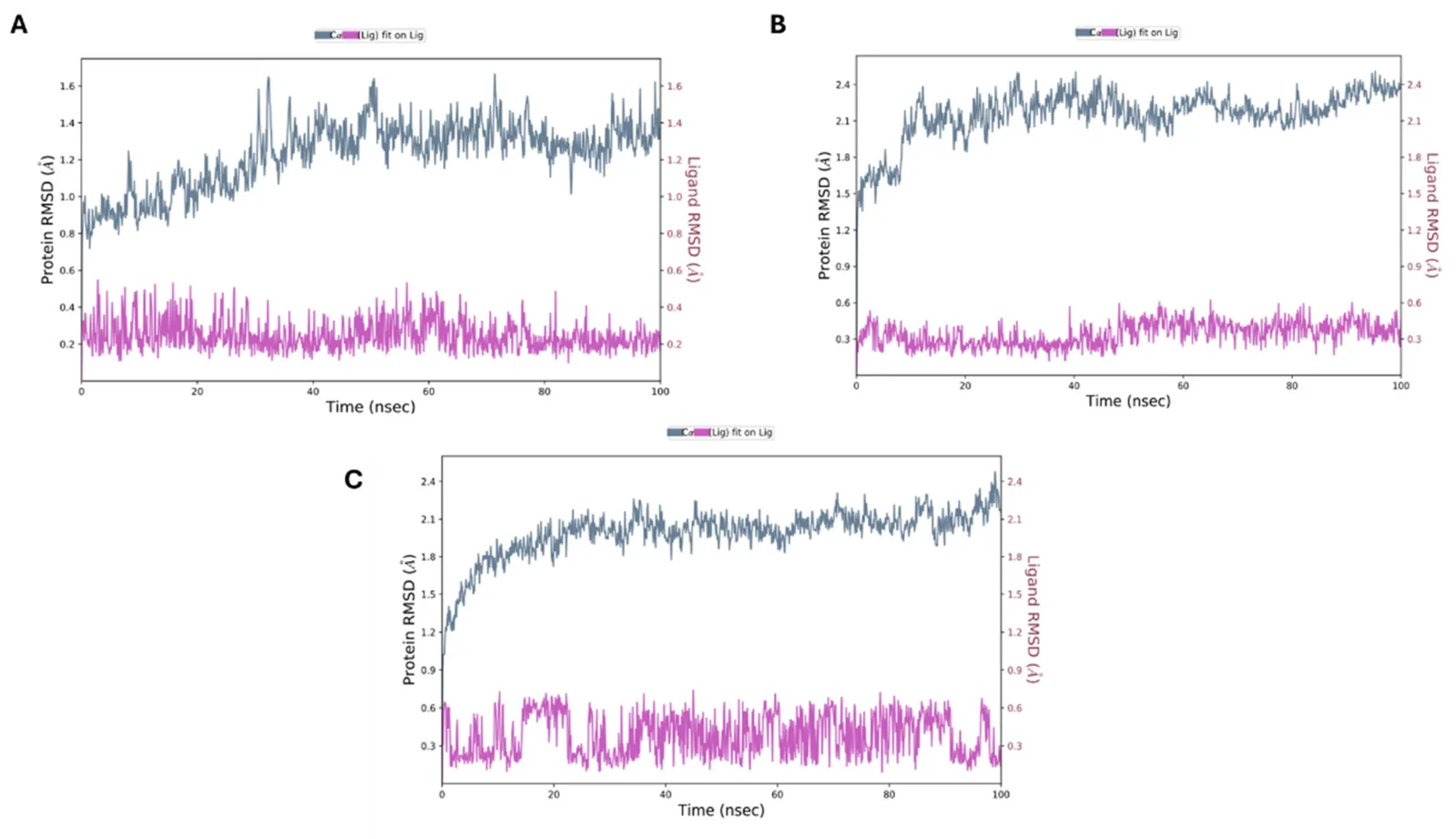
Root mean square deviation (RMSD) of protein Cα atoms (blue) and ligand heavy atoms (magenta) over 100 ns MD simulations of viridicatin in complex with Nrf2/HO-1 pathway proteins. (**A**) Viridicatin–KEAP1 complex (PDB: 4IQK). (**B**) Viridicatin–HO-1 complex (PDB: 3CZY). (**C**) Viridicatin–*NQO1* complex (PDB: 1D4A). Ligand RMSD values were computed after superposition on ligand heavy atoms.

The KEAP1 complex was the most stable of the three. Its backbone Cα RMSD settled between 0.8 and 1.4 Å and the ligand RMSD stayed below 0.4 Å for the full trajectory (Figure 10A), showing that viridicatin held its position inside the Kelch pocket. In the HO-1 complex, the backbone was more mobile (Cα RMSD 1.5 to 2.4 Å) while the ligand itself stayed close to its starting geometry (Figure 10B). The *NQO1* complex behaved differently: the backbone drifted upward without reaching a clear plateau, and the ligand trace fluctuated throughout rather than converging to a flat baseline (Figure 10C). As shown below, this restlessness was mirrored by a full turnover of contact residues and by a loss of binding free energy, so *NQO1* does not sustain a stable complex.

**Figure 11:**
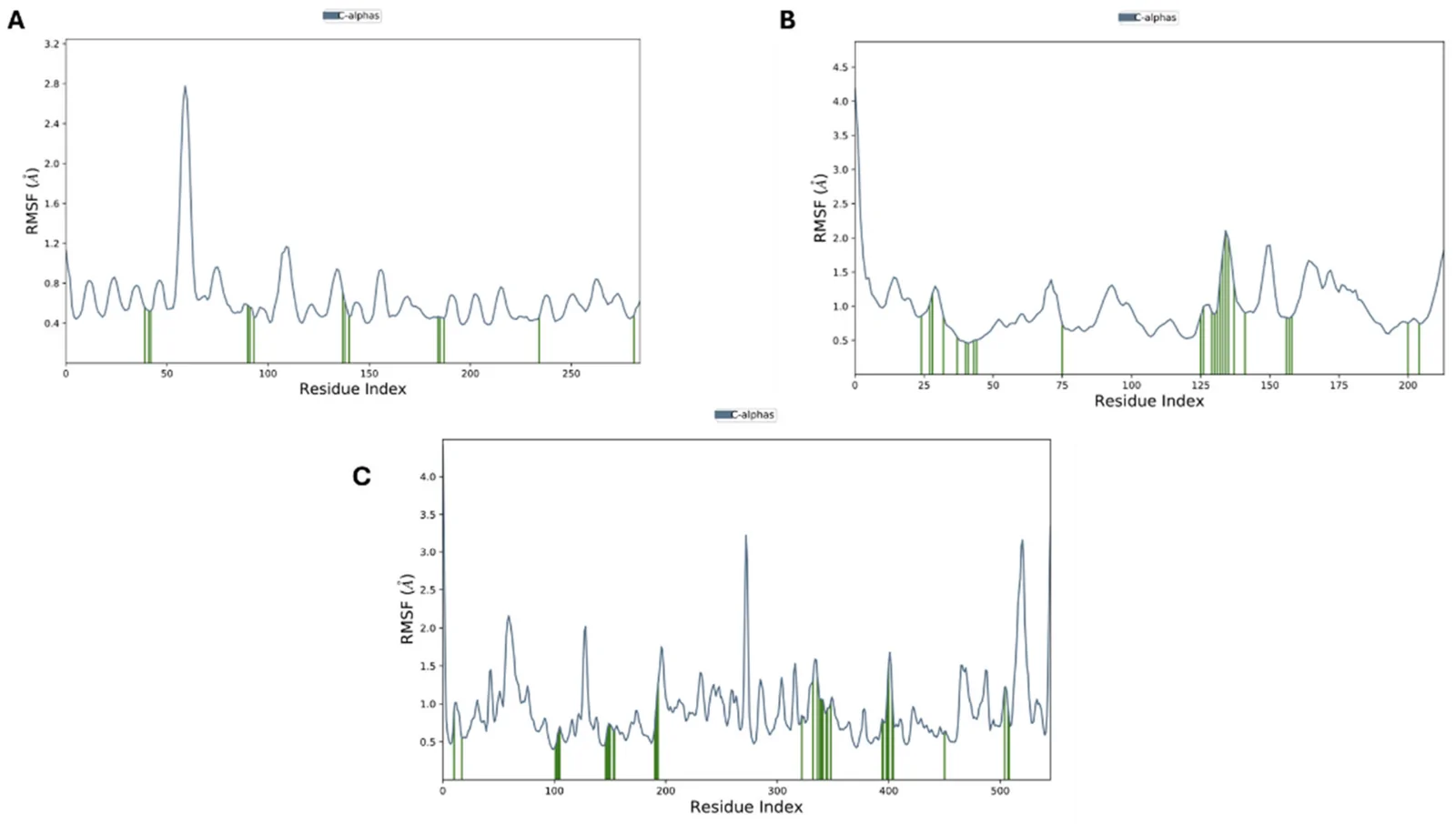
Root mean square fluctuation (RMSF) of Cα atoms for each protein during 100 ns MD simulation in complex with viridicatin. (**A**) KEAP1 (4IQK). (**B**) HO-1 (3CZY). (**C**) *NQO1* (1D4A). Green vertical bars indicate residues that formed contacts with viridicatin during the simulation.

The RMSF profiles agreed with this picture (Figure 11). In KEAP1, the binding pocket was rigid, with fluctuations below 1.2 Å across the residues that contact viridicatin (Figure 11A). The single sharp maximum near residue 60 maps to a solvent exposed surface loop far from the Nrf2 pocket and involves none of the binding residues, so it does not affect the complex. HO-1 showed raised mobility over residues 75 to 130, a set of loops lying away from the heme cavity (Figure 11B). *NQO1* fluctuated more broadly, as its largest peak (~3.0 Å) falling in a surface loop of the second monomer of the biological dimer, again outside the ligand site (Figure 11C).

**Figure 12:**
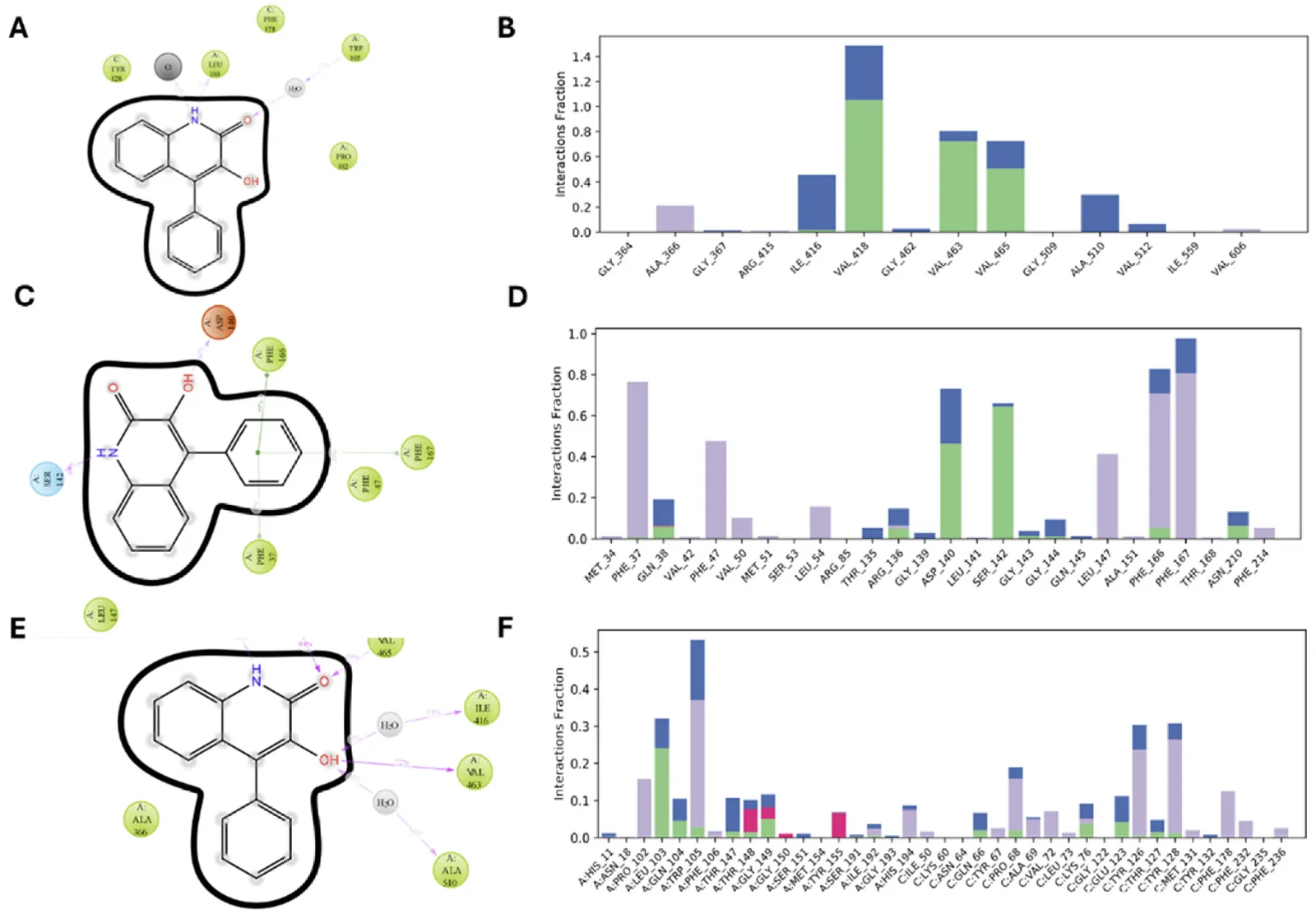
Protein–ligand interaction analysis from 100 ns MD simulations. Left panels: Two-dimensional interaction diagrams showing representative binding contacts and interaction types. Right panels: Protein–ligand contact histograms showing the fraction of simulation time during which each residue maintains interaction with viridicatin. (**A**,**B**) Viridicatin–KEAP1 (4IQK). (**C**,**D**) Viridicatin–HO-1 (3CZY). (**E**,**F**) Viridicatin–*NQO1* (1D4A). In the contact histograms, green bars indicate hydrogen bonds, purple bars hydrophobic contacts, blue bars water bridges, and magenta bars ionic interactions.

Contact analysis over the trajectory explained why the three complexes diverged (Figure 12). In KEAP1, viridicatin kept a steady set of hydrophobic contacts with Val418, Val463, Val465, Ile416, Ala366, and Ala510 (Figure 12A,B). Two of these, Ala366 and Ala510, were already present in the docking pose, while the three valines and Ile416 were recruited as the pocket relaxed, widening the interaction network. In HO-1, the contacts shifted from the docking residues (Thr26, His25, Lys22) to a neighboring cluster of aromatic residues (Phe37, Phe47, Phe166, Phe167), together with Ser142 and Asp140 (Figure 12C,D), so the ligand settled into a nearby pose held mainly by π stacking. In NQO1, the persistent contacts (Leu103, Tyr128, Phe178, Trp105, Pro102) shared nothing with the docking residues (Pro219, Tyr221, Pro224) (Figure 12E,F), confirming that the ligand had left its initial site.

Endpoint MM-GBSA energies quantified these trends at 0 and 100 ns (Table 3). KEAP1 held a strong and stable binding free energy (ΔGbind −49.13 kcal/mol at 0 ns and −42.74 kcal/mol at 100 ns), carried mainly by van der Waals and lipophilic terms. HO-1 strengthened over the run from −41.42 to −52.75 kcal/mol, indicating that the pose it adopted in the solvent was more favorable than the docking pose. *NQO1* moved the other way, weakening from −51.21 to −33.81 kcal/mol, with ligand efficiency falling from −2.85 to −1.88 kcal/mol per heavy atom. The cross-correlation and principal component analyses (Supplementary Figures S12 and S13) told the same story, with KEAP1 sampling the most compact conformational space of the three.

Taken together, the simulations mark KEAP1 as the stable primary partner of viridicatin, keeping HO-1 as a credible secondary interaction and ruling out a durable *NQO1* complex, which is in line with the docking ranking and with the cellular results.

### 3.11. Physicochemical and ADMET Profile of Viridicatin

**Figure 13:**
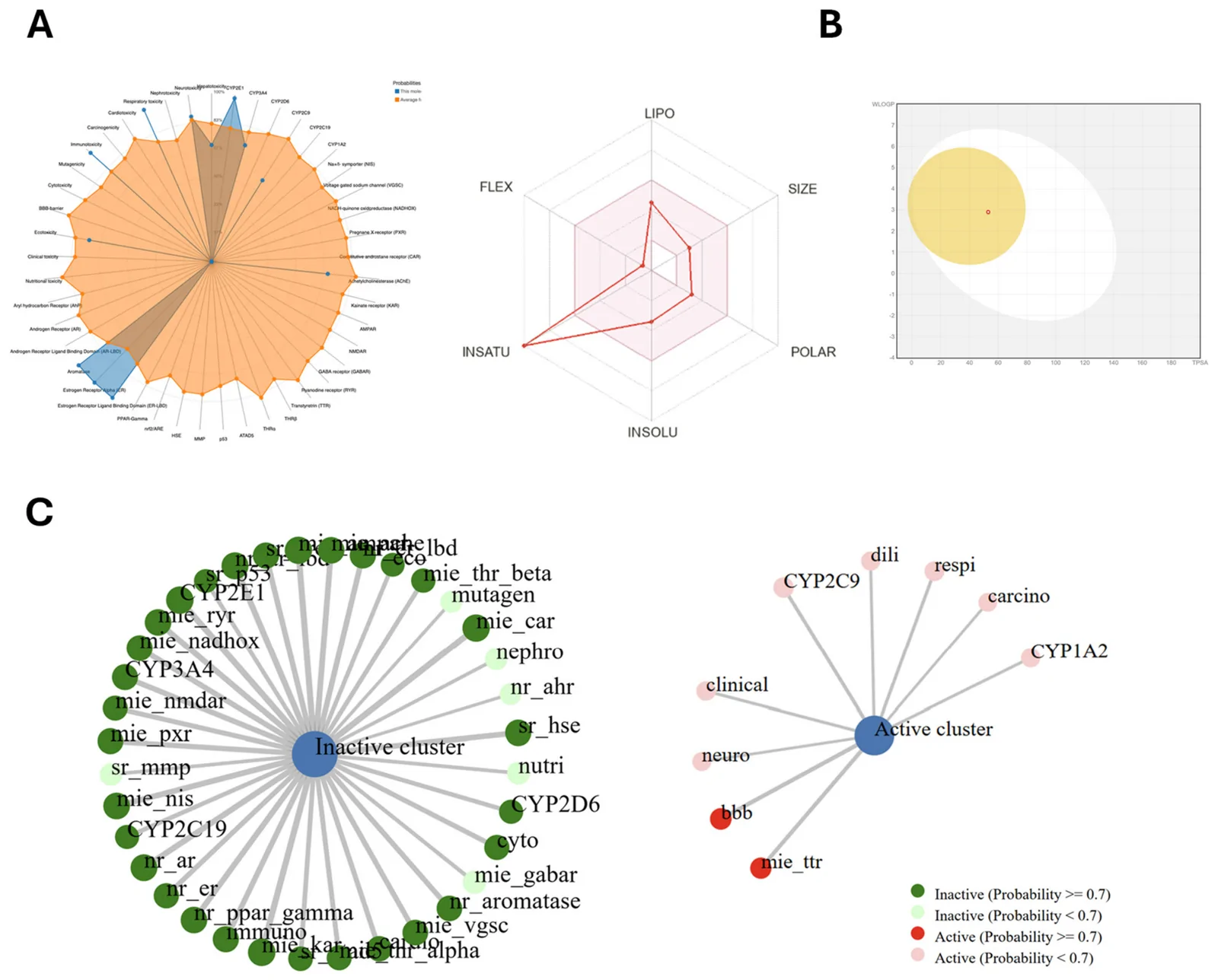
In silico ADMET profiling and drug-likeness evaluation of viridicatin. (**A**) Bioavailability radar plot showing viridicatin’s physicochemical properties (colored zone) against drug-like boundaries. The pink shaded area represents the optimal range for oral bioavailability. LIPO: lipophilicity (−0.7 < XLOGP3 < +5.0); SIZE: molecular weight (150–500 Da); POLAR: polarity (20–130 Å^2^); INSOLU: insolubility (log S > −6); INSATU: insaturation (Fraction Csp3 > 0.25); FLEX: flexibility (rotatable bonds < 9). (**B**) BOILED-Egg prediction model for viridicatin showing predicted gastrointestinal absorption (white region) and blood–brain barrier penetration (yellow region). Viridicatin (blue point) is positioned within the yellow region, indicating predicted BBB permeability and non-P-glycoprotein substrate status. (**C**) ProTox-3.0 toxicity prediction dashboard for viridicatin displaying predicted LD_50_, toxicity class, organ toxicity endpoints, Tox21 nuclear receptor and stress response pathway predictions, and metabolism (CYP) predictions. Biological activity network visualization showing the predicted active (probability ≥ 0.7, red/orange nodes) and inactive (probability < 0.7, green nodes) pathways for viridicatin. The active cluster identifies BBB penetration and selective CYP interactions, while the inactive cluster demonstrates minimal interaction with nuclear receptors, neurotransmitter receptors, and the Nrf2/ARE pathway, supporting an indirect mechanism of Nrf2 activation via KEAP1 binding.

The drug likeness and pharmacokinetic profile were examined to judge whether viridicatin is a reasonable starting point for a central nervous system agent (Table 4; Figure 13). The molecule (C_15_H_11_NO_2_; 237.25 Da) met the Lipinski, Ghose, Veber, Egan, and Muegge rules without any violations. Its moderate lipophilicity (consensus log P 2.70) and small polar surface area (53.09 Å^2^) sit within the range associated with passive brain penetration, and the BOILED-Egg model placed it in the region predicting blood–brain barrier permeability without P glycoprotein efflux (Figure 13). Metabolism is predicted to run mainly through CYP1A2 and CYP3A4, with a short half-life (0.80 h) and a low hERG liability (0.162). In the ProTox assessment, the compound fell in toxicity to class 4 (LD_50_ 1000 mg/kg) and was predicted to be inactive for cardiotoxicity, immunotoxicity, and cytotoxicity, agreeing with the absence of measurable toxicity in HMC-3 cells up to 100 µM.

One prediction is directly relevant to the proposed mechanism. Viridicatin was inactive in the Nrf2/ARE reporter pathway (SR-ARE; 0.89), meaning that it is not expected to switch on Nrf2 by covalent or electrophilic modification. This fits the docking and simulation results showing that the compound binds the KEAP1 Kelch surface rather than Nrf2 itself, and supports an indirect route to Nrf2 activation through the KEAP1–Nrf2 interface. Predicted inactivity at the androgen, estrogen, aromatase, and PPARγ endpoints indicate a clean off-target profile. These values are computational estimates and are presented as a prioritization aid rather than as measured pharmacokinetics.

## 4. Discussion

The genus *Penicillium* is known to produce a wide variety of bioactive secondary metabolites with applications in medicine, industry, and agriculture. Among these, viridicatin stands out for its unique chemical structure and for some of its reported biological activities [36]. Viridicatin belongs to the group of quinoline-derived alkaloids, and has demonstrated antimicrobial, antifungal, and antioxidant activities [37,38]. Viridicatin was first isolated from the fungus *Penicillium viridicatum*, but no structural formula was proposed at that time [39]. Later, the molecular structure of viridicatin and its biosynthesis were elucidated [40]. More recently, viridicatin was isolated, inter alia, from *Penicillium commune* XL-0473, and it was found as a mycotoxin contaminant in total mixed rations used for feeding cows in dairy farms [41,42]. The presence of viridicatin in *Penicillium* cultures suggests an ecological role in the fungus’s defense against competing microorganisms in its natural habitat. Antibiotic activity of viridicatin against *Mycobacterium tuberculosis* was reported in 2015 [43]. Furthermore, viridicatin’s inhibitory activity on the expression of matrix metalloproteinases MMP-2 and MMP-9 in HT1080 cells has been reported [44]. However, no previous reports of its activity in cell protection have been found for this molecule.

Neurons normally depend on microglia to sustain them through a variety of mechanisms. A reduction in the numbers and support functions of microglia due to senescence contributes to the development of neurodegenerative changes and synapse loss [45]. One of the factors that affects both the numbers and functions of microglia is oxidative stress. An excess of ROS, accompanied by a reduction in the capacity of natural antioxidant systems, leads to cell dysfunction and death [46]. Prolonged or persistent exposure to non-physiological levels of oxidative stress and ROS has been shown to be associated with a variety of diseases, especially neurodegenerative diseases [9,47]. The CNS exhibits very high oxygen turnover combined with a generally weak antioxidant system. Nerve tissue spontaneously generates and releases H_2_O_2_ into the extracellular/cerebrospinal fluid, which is poor in antioxidants. The concentration of H_2_O_2_ could reach up to 1 mM in the cerebrospinal fluid under physiological conditions, while in neurodegeneration, production is promoted through several different pathways. These conditions can be mimicked in vitro by exogenous administration of H_2_O_2_ [48]. As expected, H_2_O_2_ exposure decreased the viability and proliferation of HMC-3 microglial cells and disrupted their normal morphology. Pre-treatment with viridicatin at 100 µM effectively counteracted these effects, restoring viability to near-control levels while exhibiting minimal intrinsic cytotoxicity at concentrations up to 100 µM (Figure 2). These results establish viridicatin as a cytoprotective agent in this model and are consistent with the neuroprotective properties reported for other quinoline-derived compounds [49]. Furthermore, viridicatin prevented H_2_O_2_-induced mitochondrial membrane depolarization after 6 h of oxidative challenge, in agreement with previous reports demonstrating that mitochondrial dysfunction is an early event in oxidative stress-mediated cell death [50]. The results obtained demonstrate that viridicatin protected HMC-3 microglial cells from mitochondrial membrane depolarization six hours after treatment with H_2_O_2_ (300 µM).

The activation of the Nrf2 pathway is one of the main mechanisms protecting cells against oxidative damage by regulating endogenous antioxidant molecules [51,52].

Many studies have reported that Nrf2 activates and increases its presence in the nucleus when oxidative stress occurs [53]. In this study, H_2_O_2_ induced a marked accumulation of Nrf2 in the nucleus, consistent with the activation of an adaptive antioxidant response to a strong oxidative challenge. Viridicatin alone also increased nuclear Nrf2, although to a lesser extent, suggesting a moderate activation or priming of endogenous antioxidant defenses under basal conditions. Notably, treatment with viridicatin and H_2_O_2_ restored nuclear Nrf2 abundance toward control values (Figure 5A,B). This reduction relative to H_2_O_2_ alone should not necessarily be interpreted as inhibition of Nrf2 signaling. Rather, together with the preservation of mitochondrial function and reduction in cell death observed in the present study, it may indicate that viridicatin attenuated the oxidative burden and promoted the recovery of redox homeostasis, thereby reducing the requirement for sustained Nrf2 nuclear accumulation, However, because Nrf2 was evaluated at a single time point, further time-course studies are needed to clarify the kinetics and underlying mechanism of this response. In parallel, viridicatin increased HO-1 protein abundance and induced *NQO1* expression at the transcriptional level, although the latter response was not accompanied by a significant increase in *NQO1* protein abundance at 6 h (Figure 6 and Figure 7). Although HO-1 is commonly known as an antioxidant enzyme located in the cytoplasm, accumulated evidence has shown that HO-1 undergoes proteolytic cleavage from the ER and translocate to other organelles, including caveolae, mitochondria, and nuclei in response to stress stimuli [54]. However, some authors have suggested that the nuclear translocation of HO-1 could be involved in a cell preservation mechanism under oxidative stress conditions, leading to survival advantages for cancer progression [55,56,57]. On the other hand, *NQO1* is a pleiotropic enzyme that reduces ubiquinone and vitamin E quinone to antioxidant forms, suggesting a primary protective role [58].

Viridicatin did not significantly increase the abundance of the *NQO1* protein at 6 h; however, treatment with viridicatin alone increased *NQO1* mRNA levels, and this transcriptional response was further enhanced by combined treatment with H_2_O_2_. The discrepancy between the mRNA and *NQO1* protein responses may reflect a time lag between transcriptional induction and the translation and accumulation of the corresponding protein. Indeed, mRNA abundance does not necessarily predict protein abundance at any given time, particularly during dynamic cellular responses, as protein levels are also influenced by mRNA stability, translation efficiency, and protein turnover [59,60]. Therefore, these observations support an early transcriptional induction of *NQO1* consistent with modulation of the Nrf2/ARE pathway, although no corresponding increase in *NQO1* protein was detected at the time point assessed.

PBMCs from individuals with mild cognitive impairment display heightened susceptibility to H_2_O_2_-induced cell death compared with cells from cognitively healthy age-matched controls, supporting their use as an accessible translational model. In the present study, viridicatin significantly attenuated the loss of viability induced by 100 µM H_2_O_2_ in PBMCs from MCI donors, partially restoring cell survival. However, at 300 µM H_2_O_2_, the protective effect was overwhelmed, suggesting that viridicatin’s capacity to counteract oxidative damage is limited by the severity of the insult. Notably, in PBMCs from cognitively healthy donors, a lower concentration of viridicatin (50 µM) was sufficient to abolish the cytotoxic effect of 300 µM H_2_O_2_, preserving viability at levels comparable to untreated cells. This differential response likely reflects the diminished baseline antioxidant buffering capacity, which may require higher concentrations of a protective agent to achieve equivalent rescue. Taken together, these findings extend the neuroprotective profile of viridicatin beyond immortalized cell lines to clinically relevant primary human cells and suggest that the compound’s efficacy is modulated by the intrinsic redox status of the target cell population.

Molecular docking analyses provided a structural rationale for the observed biological effects, identifying KEAP1 as the most favorable target (−8.0 kcal/mol) with viridicatin occupying the β-propeller pocket that mediates the KEAP1–Nrf2 protein–protein interaction. By competing with Nrf2 at this interface, viridicatin may stabilize Nrf2 under basal conditions and prime the cellular antioxidant defense before the oxidative insult arrives. This is consistent with the reduced Nrf2 nuclear accumulation observed in viridicatin/H_2_O_2_ co-treated cells relative to H_2_O_2_ alone. The Tox21 prediction that viridicatin is inactive for the Nrf2/ARE stress response pathway (SR-ARE; probability 0.89) further supports an indirect mechanism, ruling out direct electrophilic activation of Nrf2. MD simulations supported the docking findings, as the viridicatin–KEAP1 complex remained stable throughout 100 ns of simulation (ligand RMSD < 0.4 Å), with MM-GBSA binding free energy of −42.74 kcal/mol at equilibrium and an expanded hydrophobic network involving Val418, Val463, Val465, and Ile416 reinforcing the docking contacts at Ala366 and Ala510. For HO-1, the ligand adopted an alternative binding mode dominated by π-stacking with Phe37, Phe166, and Phe167, yielding an improved binding free energy of −52.75 kcal/mol at equilibrium; this favorable interaction may contribute to the prevention of non-canonical HO-1 nuclear translocation observed in co-treated cells. In contrast, viridicatin failed to maintain a stable complex with NQO1, shown by a complete turnover of contact residues over the trajectory (Figure 12E,F) and a marked decline in binding free energy (ΔGbind from −51.21 to −33.81 kcal/mol), confirming that *NQO1* is not a direct target and that changes in its expression arise from upstream redox normalization through the KEAP1–Nrf2 axis. Although these computational predictions require experimental confirmation through binding assays and site-directed mutagenesis, the convergence of docking, MD, and MM-GBSA data with the in vitro cellular phenotypes provides a coherent mechanistic framework for viridicatin’s neuroprotective action.

## Figures and Tables

**Table 1:** Demographics from patients included in viridicatin in vitro assays on PBMC.

	Control (*n* = 4)	aMCI/Mild AD (*n* = 8)
Sex (% female)	4 (100%)	5 (62.5%)
Age (mean ± SE) Range	74.3 ± 4.3 69–87	70.4 ± 2.8 53–77
MoCA test score (mean ± SE) Range	28.3 ± 1.7 26–30	16.5 ± 1.7 11–23

MCI: mild cognitive impairment; aMCI: amnestic-type mild cognitive impairment; AD: Alzheimer’s disease.

**Table 2:** Molecular docking results of viridicatin with Nrf2/HO-1 pathway proteins.

Ligand	Target Receptor (PDB ID)	Binding Affinity (kcal/mol)	Interacting Amino Acids	Interaction Types	H-Bonds
Vir	KEAP1 Kelch Domain (4IQK)	−8.0	Gly464, Val512, Ala366, Ala510	Conventional H-Bond, Carbon H-Bond, Alkyl	1
Vir	HO-1 (3CZY)	−7.5	Thr26, His25, Lys22, Lys18, Hem300	Conventional H-Bond, Pi-Cation	3
Vir	*NQO1* (1D4A)	−6.1	Pro219, Tyr221, Pro224	Conventional H-Bond, Pi-Alkyl	1

**Table 3:** MM-GBSA binding free energy analysis of viridicatin with Nrf2/HO-1 pathway proteins at 0 ns and 100 ns of MD simulations.

Target	Frame	ΔG_bind	ΔG_Coulomb	ΔG_Hbond	ΔG_Lipo	ΔG_vdW	ΔG_Solv	LE
KEAP1 (4IQK)	0 ns	−49.13	−7.92	−0.37	−18.35	−39.39	16.58	−2.73
KEAP1 (4IQK)	100 ns	−42.74	−9.75	−0.43	−14.87	−34.77	16.08	−2.37
HO-1 (3CZY)	0 ns	−41.42	−3.84	−0.23	−23.39	−27.77	14.83	−2.30
HO-1 (3CZY)	100 ns	−52.75	−11.33	−0.61	−21.67	−32.51	14.06	−2.93
*NQO1* (1D4A)	0 ns	−51.21	−13.65	−0.26	−23.74	−35.36	23.58	−2.85
*NQO1* (1D4A)	100 ns	−33.81	−7.70	−0.23	−16.34	−20.16	12.50	−1.88

All energy values are in kcal/mol. LE: ligand efficiency (ΔGbind/number of heavy atoms); ΔG_Coulomb: Coulomb interaction energy; ΔG_Hbond: hydrogen bond energy; ΔG_Lipo: lipophilic energy; ΔG_vdW: van der Waals energy; ΔG_Solv: solvation energy (Generalized Born).

**Table 4:** Physicochemical properties and Lipinski parameters of viridicatin.

Absorption and Distribution	Metabolism	Medicinal Chemistry
**BBB**: Yes	CYP1A2 inhibitor: Yes (+++)	Lipinski Rule: Yes
**HIA**: High	CYP1A2 substrate: Yes (+++)	Pfizer Rule: Yes
**Caco-2 Permeability**: −5.02	CYP2C19 inhibitor: No (−−−)	GSK Rule: Yes
**P-gp Substrate**: No (−)	CYP2C19 substrate: No (−−−)	Golden Triangle: Yes
**P-gp Inhibitor**: No (−−)	CYP2C9 inhibitor: No (−−−)	PAINS: 0
**PPB**: 97.9%	CYP2C9 substrate: No (−−−)	Brenk Alerts: 0
**VDss**: −0.28 L/kg	CYP2D6 inhibitor: No (−−−)	SA Score: 2.00 (Easy)
	CYP2D6 substrate: No (−−−)	QED: 0.683
**Excretion**	CYP3A4 inhibitor: No (−−−)	Bioavailability Score: 0.55
**Clearance**: 5.01 mL/min/kg	CYP3A4 substrate: Yes (+++)	
**Half-Life**: 0.80 h	HLM stability: Stable (−−−)	
**Toxicity**		
**hERG Blockers**: 0.162		
**DILI**: 0.585		
**AMES Toxicity**: 0.572		

Note: Prediction probability values are denoted as: 0–0.1 (−−−), 0.1–0.3 (−−), 0.3–0.5 (−), and 0.9–1.0 (+++). Higher ‘+’ values indicate greater probability of the predicted property.

## Data Availability

The crystallographic data for viridicatin have been deposited at the Cambridge Crystallographic Data Centre under accession number CCDC2524160 and can be obtained free of charge at https://www.ccdc.cam.ac.uk (accessed on 2 June 2026). The raw NMR data and the remaining datasets generated and analyzed in this study are openly available in the Zenodo repository at https://doi.org/10.5281/zenodo.19111060 (accessed on 2 June 2026). Any further data is available from the corresponding authors upon reasonable request.

## Supplementary Materials

The following supporting information can be downloaded at: https://www.mdpi.com/article/10.3390/antiox15091089/s1. Single-crystal X-ray structure analysis of viridicatin (general details); Table S1: Crystal data and details of structure refinement for viridicatin; Table S2: ^13^C (500 MHz) and ^1^H (125 MHz)-NMR Spectroscopic data for viridicatin in acetone-*d*_6_; Figure S1: Molecular structure with crystallographic atom labeling of viridicatin. Atom labeling refers to the positional numbering used in the CIF file and differs from the numbering scheme used for NMR-data assignment. Displacement ellipsoids are shown at the 50% probability level; Figure S2: ^1^H- (red) and ^13^C-NMR-Data (green), signal assignment and HMBC interactions (blue) for viridicatin; Figure S3: ^1^H NMR (500 MHz, acetone-d_6_) of viridicatin with the full range; Figure S4: ^1^H NMR (500 MHz, acetone-d_6_) of viridicatin, 7.60–7.10 ppm; Figure S5: ^13^C{^1^H} NMR (125 MHz, acetone-d_6_) of viridicatin; Figure S6: H,H-COSY (500 MHz, acetone-d_6_) of viridicatin; Figure S7: HSQC (500/125 MHz, acetone-d_6_) of viridicatin; Figure S8: HMBC (500/125 MHz, acetone-d_6_) of viridicatin; Figure S9: NOESY (500 MHz, acetone-d_6_) of viridicatin; Figure S10: Mycoplasma testing of HMC-3 cultures before and after Plasmocin treatment; Figure S11: Viridicatin prevents the loss of viability induced by oxidative stress relative to basal viability in healthy patient-derived cells; Figure S12: Dynamic cross-correlation matrices (DCCMs) from 100 ns MD simulations of viridicatin in complex with (A) KEAP1 (4IQK), (B) HO-1 (3CZY), and (C) *NQO1* (1D4A); Figure S13: Principal component analyses (PCAs) of MD trajectories for viridicatin in complex with (A) KEAP1 (4IQK), (B) HO-1 (3CZY), and (C) *NQO1* (1D4A).
