# Supplementary material for: Viridicatin from the Antarctic Fungus Penicillium sp. Protects Human Microglia and Patient-Derived Peripheral Immune Cells Against Oxidative Stress
Source: Antioxidants (Basel). 2026 Aug 30;15(9):1089. doi: 10.3390/antiox15091089 (PMC13603828; doi:10.3390/antiox15091089)

Supplementary Information

Viridicatin from the Antarctic Fungus *Penicillium* sp. Protects Human Microglia and Patient-Derived Peripheral Immune Cells Against Oxidative Stress

Cristian Paz [1], Muhammad Javid Iqbal [1], Andrea Cristina Paula Lima [2,3], Alejandro Luarte [4,5,6], Pablo Lazcano [6], Ursula Wyneken [4,5,6], María Isabel Behrens [7,8,9,10], Daniela Ponce [7], Nicole Jeraldo [7], Sigisfredo Garnica [11], Cecilia Villegas [12], Vaderament-A. Nchiozem-Ngnitedem [13], Bernd Schmidt [13], Eric Sperlich [13], Nicole Cortez [1,*] and Viviana Burgos [14,15,*]

[1] Laboratory of Natural Products & Drug Discovery, Department of Basic Sciences, Faculty of Medicine, Universidad de La Frontera, Temuco 4780000, Chile; cristian.paz@ufrontera.cl (C.P.); m.iqbal01@ufromail.cl (M.J.I.)
[2] Biomedical Neuroscience Institute, Faculty of Medicine, University of Chile, Santiago 8380453, Chile; acpaulalima@u.uchile.cl
[3] Institute for Research in Dental Sciences, Faculty of Dentistry, University of Chile, Santiago 8380453, Chile
[4] Faculty of Medicine, Universidad de los Andes, Santiago 7620001, Chile; aluarte@uandes.cl (A.L.); uwyneken@uandes.cl (U.W.)
[5] Program in Neuroscience, Center for Biomedical Research, and Innovation (CiiB), Universidad de los Andes, Santiago 7620001, Chile
[6] IMPACT—Center of Interventional Medicine for Precision and Advanced Cellular Therapy, Santiago 7620001, Chile; plazcano@uandes.cl
[7] Center for Advanced Clinical Research (CICA), Clinical Hospital of the University of Chile, Santiago 8380447, Chile; behrensl@uchile.cl (M.I.B.); dponcedelavega@gmail.com (D.P.); nicole.jeraldo@ug.uchile.cl (N.J.)
[8] Department of Neuroscience, Faculty of Medicine, University of Chile, Santiago 8380453, Chile
[9] Department of Neurology and Neurosurgery, Clinical Hospital of the University of Chile, Santiago 8380447, Chile
[10] Department of Neurology and Psychiatry, German Clinic of Santiago-University of Development, Santiago 7650568, Chile
[11] Laboratorio de Micología, Instituto de Bioquímica y Microbiología, Universidad Austral de Chile, Valdivia 5090000, Chile; sigisfredo.garnica@uach.cl
[12] Departamento de Ciencias Biológicas y Químicas, Facultad de Recursos Naturales, Universidad Católica de Temuco, Temuco 4780000, Chile; cecilia.villegas@uct.cl
[13] Institut für Chemie, Universität Potsdam, Karl-Liebknecht-Str. 24-25, D-14476 Potsdam, Germany; vaderament-alexe.nchiozem-ngnitedem@uni-potsdam.de (V.-A.N.-N.); bernd.schmidt@uni-potsdam.de (B.S.); eric.sperlich@uni-potsdam.de (E.S.)
[14] Escuela de Tecnología Médica, Facultad de Salud, Universidad Santo Tomás, Temuco 4780000, Chile
[15] Centro de Investigación en Prevención y Cuidados de la Salud (+SALUD), Facultad de Salud, Universidad Santo Tomás, Temuco 4780000, Chile
* Correspondence: n.cortez01@ufromail.cl (N.C.); vburgos7@santotomas.cl (V.B.); Tel.: +56-9-71090520 (N.C.); +56-9-83111126 (V.B.)

A Single crystal X-ray structure analysis of viridicatin

General details of X-ray structure analysis

The crystal structure was determined by single crystal structure analysis. A suitable single crystal was selected using a Leica M205C light microscope and separated with oil. X-ray crystal structure analysis was performed on a Stadivari diffractometer (Stoe) with monochromated Mo-*Kα* radiation (λ = 0.71073 Å). The data correction was performed using the program X-Area.[1] The structure was solved by direct methods and refined against F^2 on all data by full-matrix least-squares using the SHELX suite of programs.[2,3] All non-hydrogen atoms were refined anisotropically; the hydrogen atoms were placed on calculated positions. **Table S1** was created using FinalCif.[4] The crystal structure was visualized with Mercury.[5] The data (viridicatin (**1**): CCCD 2524160) can be obtained free of charge from the Cambridge Crystallographic Data Centre, http://www.ccdc.cam.ac.uk.

(1) STOE & Cie GmbH (2018) X-Area. software package for collecting single-crystal data on STOE area-detector diffractometers, for image processing, for the correction and scaling of reflection intensities and for outlier rejection. STOE & Cie GmbH, Darmstadt.

(2) Sheldrick, G. Crystal structure refinement with SHELXL. *Acta Cryst. C* **2015**, *C71*, 3-8.

(3) Sheldrick, G. A short history of SHELX. *Acta Cryst. A* **2008**, *A64*, 112-122.

(4) FinalCif. https://dkratzert.de/finalcif.html.

(5) Macrae, C. F.; Sovago, I.; Cottrell, S. J.; Galek, P. T. A.; McCabe, P.; Pidcock, E.; Platings, M.; Shields, G. P.; Stevens, J. S.; Towler, M.; Wood, P. A. Mercury 4.0: from visualization to analysis, design and prediction. *J. Appl. Cryst.* **2020**, *53*, 226-235.

Crystallographic Data:

Table S1. Crystal data and details of structure refinement for viridicatin.

Compound	Viridicatin (**1**)
CCDC number	2524160
Empirical formula	$C_{15}H_{11}NO_2$
Formula weight	237.25
Temperature [K]	210
Crystal system	monoclinic
Space group (number)	$P2_1/n$ (14)
a [Å]	5.7785(5)
b [Å]	20.9663(18)
c [Å]	9.8243(8)
α [°]	90
β [°]	105.660(7)
γ [°]	90
Volume [$Å^3$]	1146.07(17)
Z	4
ρ_{calc} [gcm^{-3}]	1.375
μ [mm^{-1}]	0.091
F(000)	496
Crystal size [mm^3]	0.300×0.500×0.800
Crystal colour	colorless
Crystal shape	long needle
Radiation	Mo K_α<br>(λ=0.71073 Å)
2θ range [°]	5.80 to 66.07<br>(0.65 Å)
Index ranges	$-6 \leq h \leq 8$<br>$-30 \leq k \leq 31$<br>$-14 \leq l \leq 14$
Reflections collected	20388
Independent reflections	4059<br>R_{int} = 0.0209<br>R_{sigma} = 0.0142
Completeness to θ = 25.242°	99.8 %
Data / Restraints / Parameters	4059 / 0 / 166
Absorption correction T_{min}/T_{max} (method)	0.8671 / 0.9629<br>(sphere)
Goodness-of-fit on F^2	1.079
Final R indexes<br>[I≥2σ(I)]	R_1 = 0.0464<br>wR_2 = 0.1370
Final R indexes<br>[all data]	R_1 = 0.0548<br>wR_2 = 0.1462
Largest peak/hole [$eÅ^{-3}$]	0.38/−0.20

Visualization of the crystal structure and molecular structure for viridicatin.

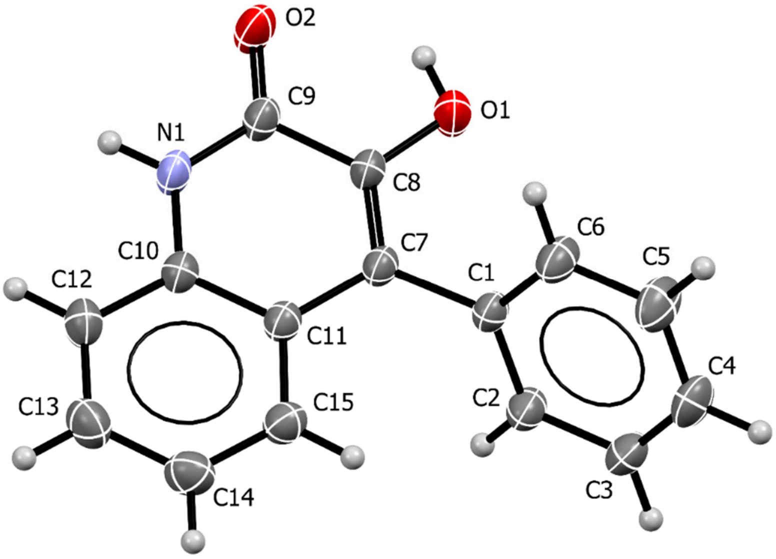

Figure S1: Molecular structure with crystallographic atom labeling of viridicatin. Atom labeling refers to the positional numbering used in the CIF file and differs from the numbering scheme used for NMR-data assignment. Displacement ellipsoids are shown at the 50% probability level.

Table S2. ^{13}C (500 MHz) and ^{1}H (125 MHz)-NMR Spectroscopic data for viridicatin in acetone-d_6.

	Viridicatin	
	$\delta_{C,\ Type}$	$\delta_{H,}$ *mult* (*J* in Hz)
**1**	-	7.92 *s* (br., NH)*
**2**	159.4, C	-
**3**	143.0, C	11.28 *s* (br., OH)*
**4**	124.7, C	-
**4a**	122.3, C	-
**5**	125.8, CH	7.22 *dd* (8.2, 1.3)
**6**	123.4, CH	7.14 *ddd* (8.2, 7.2, 1.0)
**7**	127.7, CH	7.38 *ddd* (8.2, 7.2, 1.4)
**8**	116.3, CH	7.47 *dm* (8.2)
**8a**	134.3, C	-
**1′**	134.7, C	-
**2′/6′**	130.9, CH	7.44 *m*
**3′/5′**	129.3, CH	7.55 *ddm* (7.5, 7.5)
**4′**	128.7, CH	7.47 *m*

Figure S2: ^{1}H- (red) and ^{13}C-NMR-Data (green), signal assignment and HMBC interactions (blue) for viridicatin.

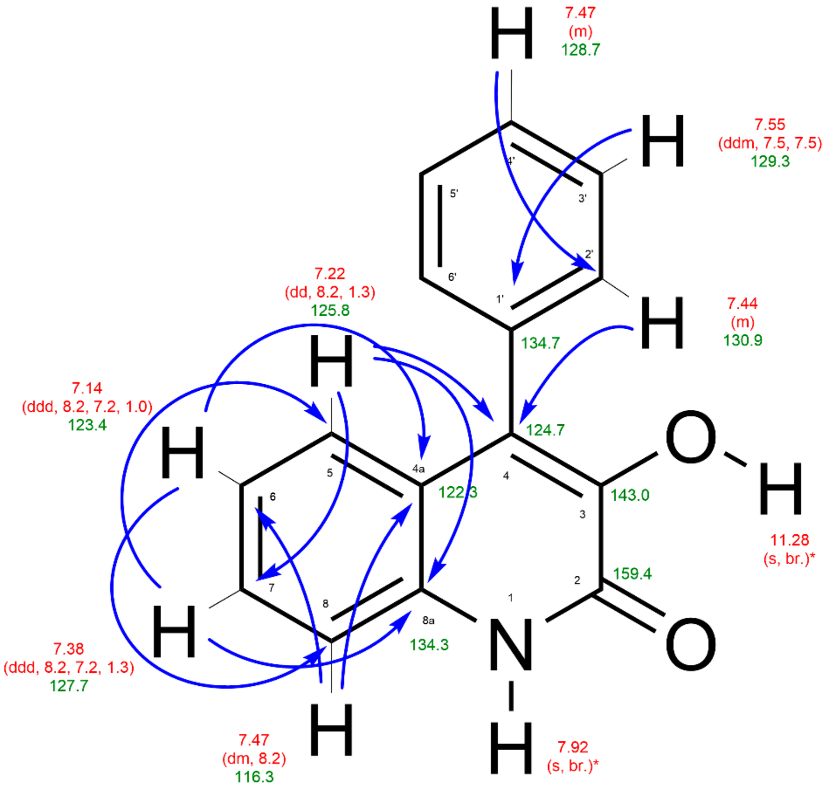

Figure S3: ^{1}H NMR (500 MHz, acetone-d_6) of viridicatin. Full range.

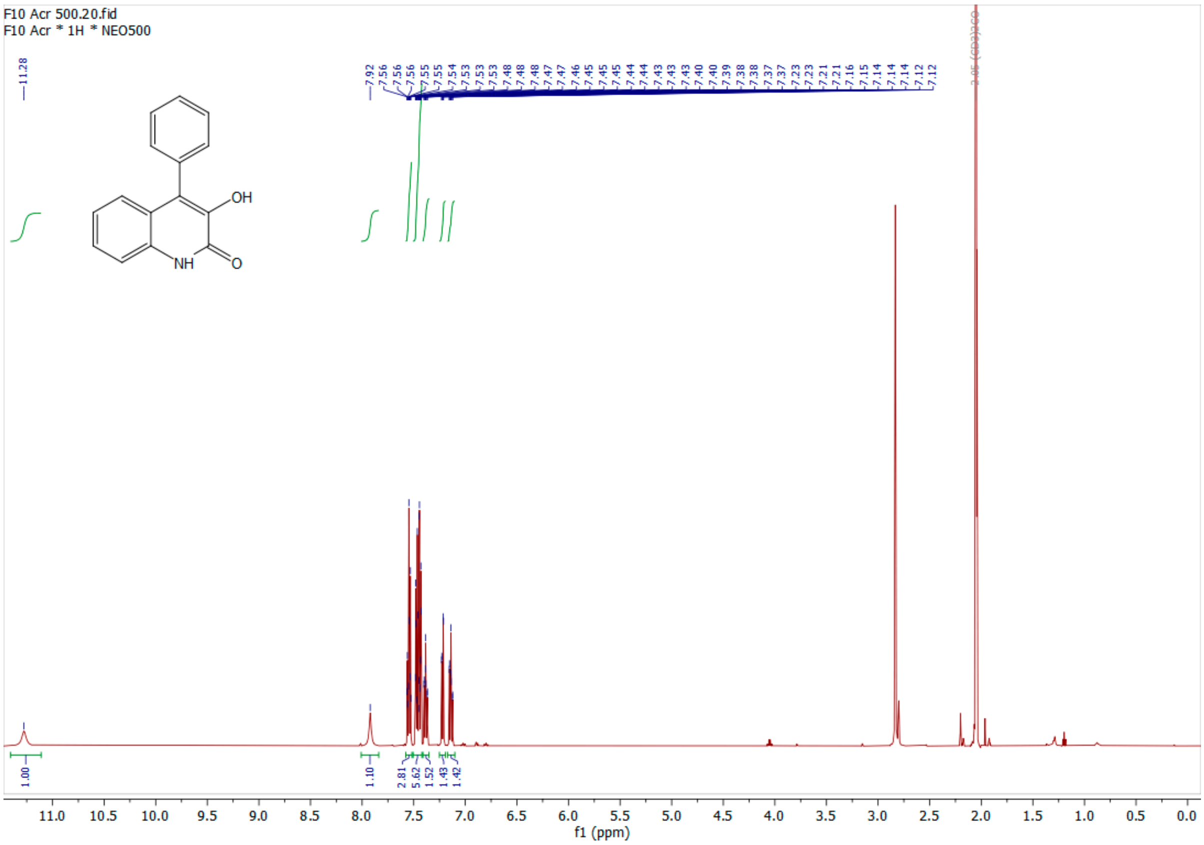

Figure S4: ^{1}H NMR (50 MHz, acetone-d_6) of viridicatin. Range from 7.60 to 7.10 ppm

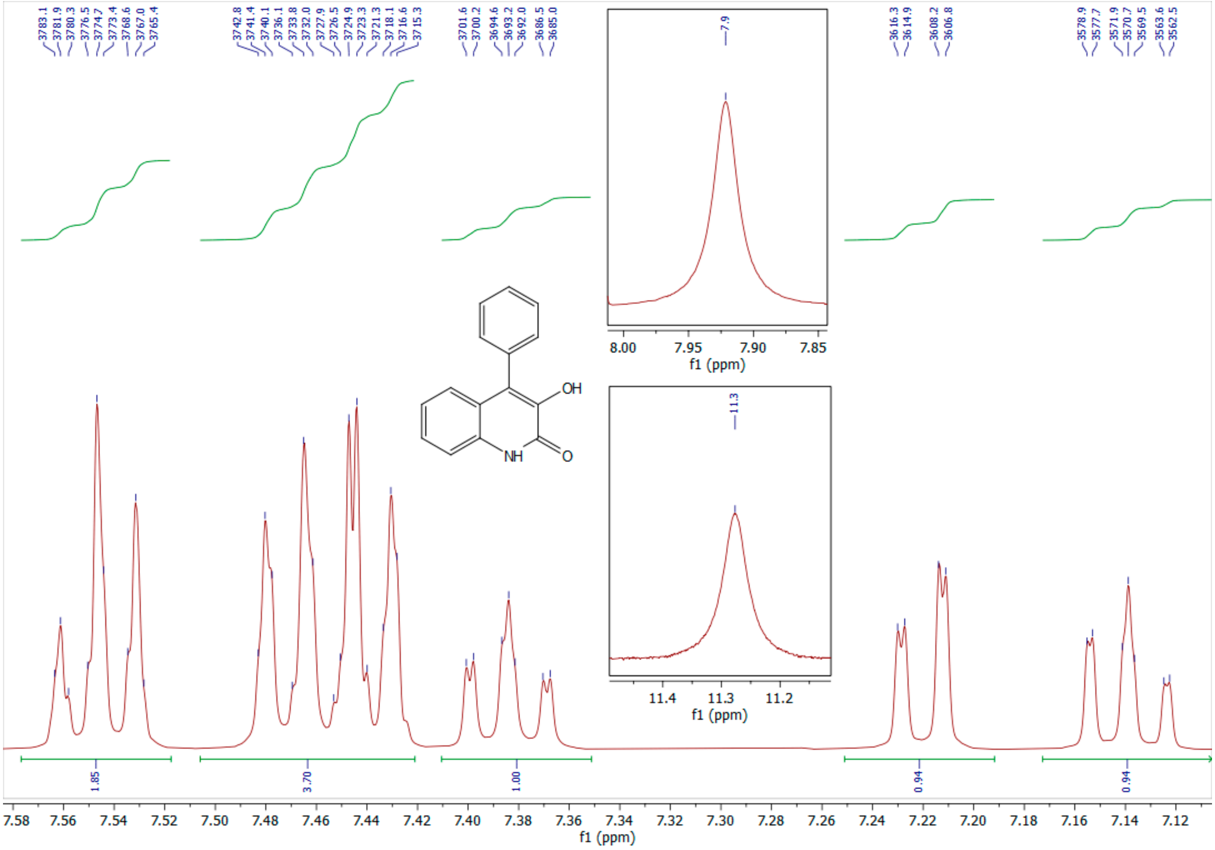

Figure S5: ^{13}C{^{1}H} NMR (125 MHz, acetone-d_6) of viridicatin.

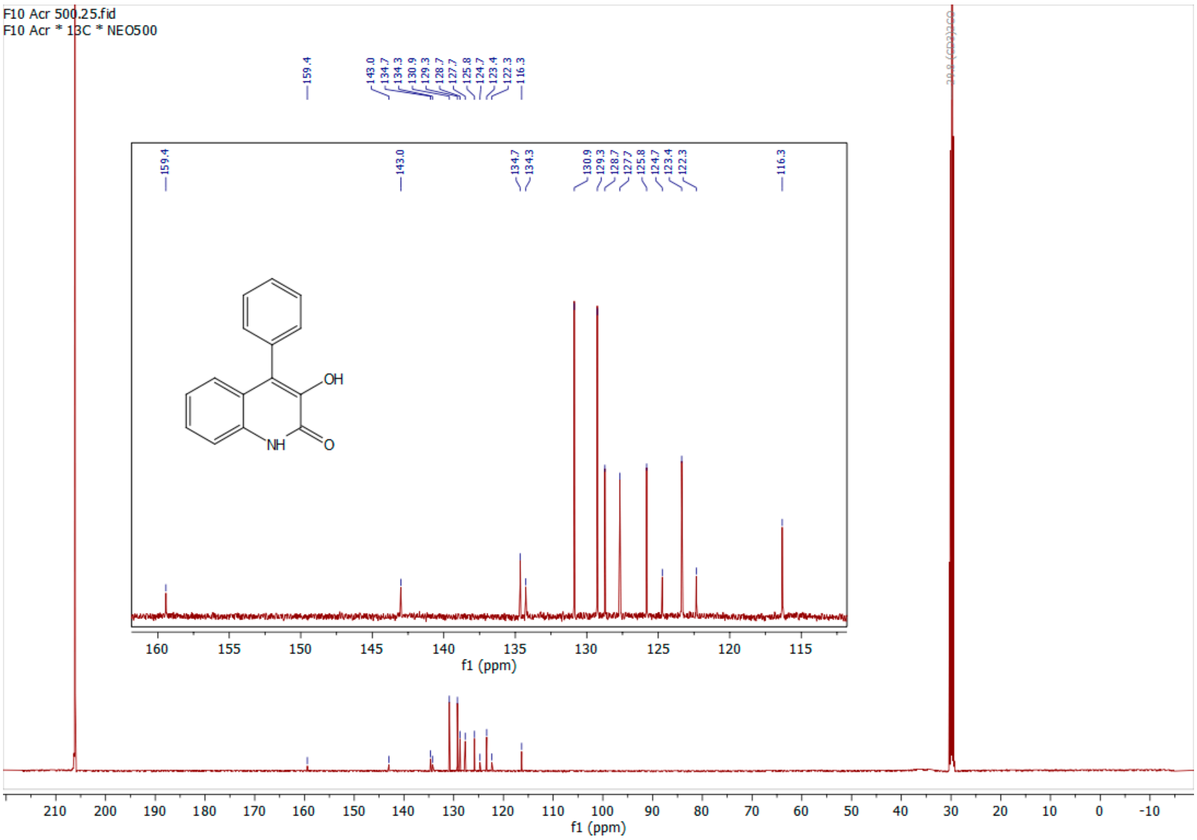

Figure S6: H,H-COSY (500 MHz, acetone-d_6) of viridicatin.

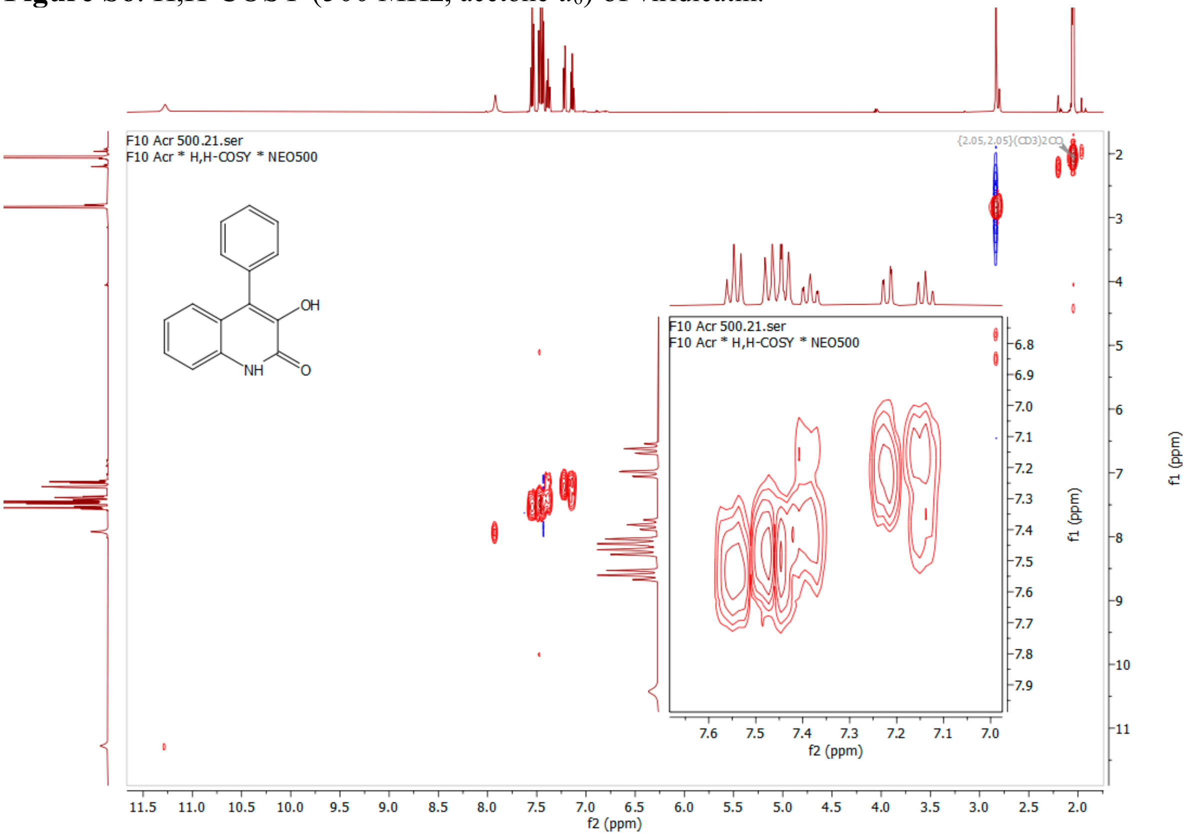

Figure S7: HSQC (500/125 MHz, acetone-d_6) of viridicatin.

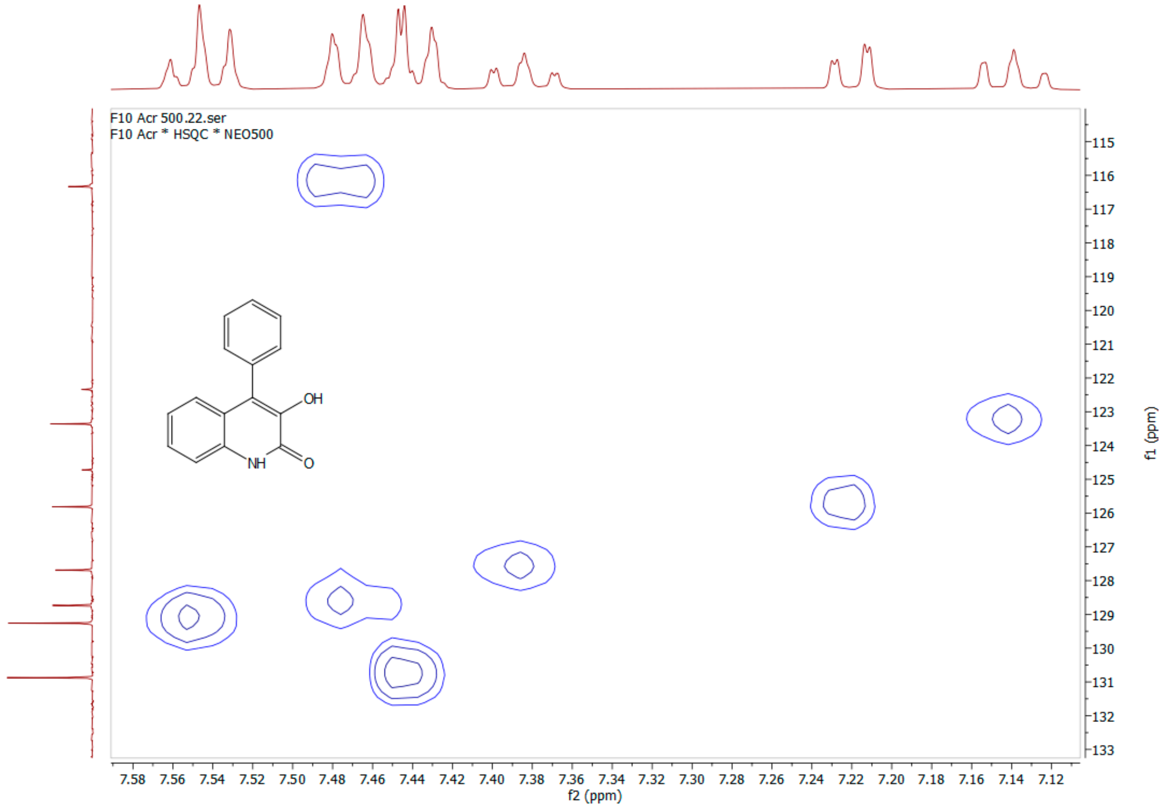

Figure S8: HMBC (500/125 MHz, acetone-d_6) of viridicatin.

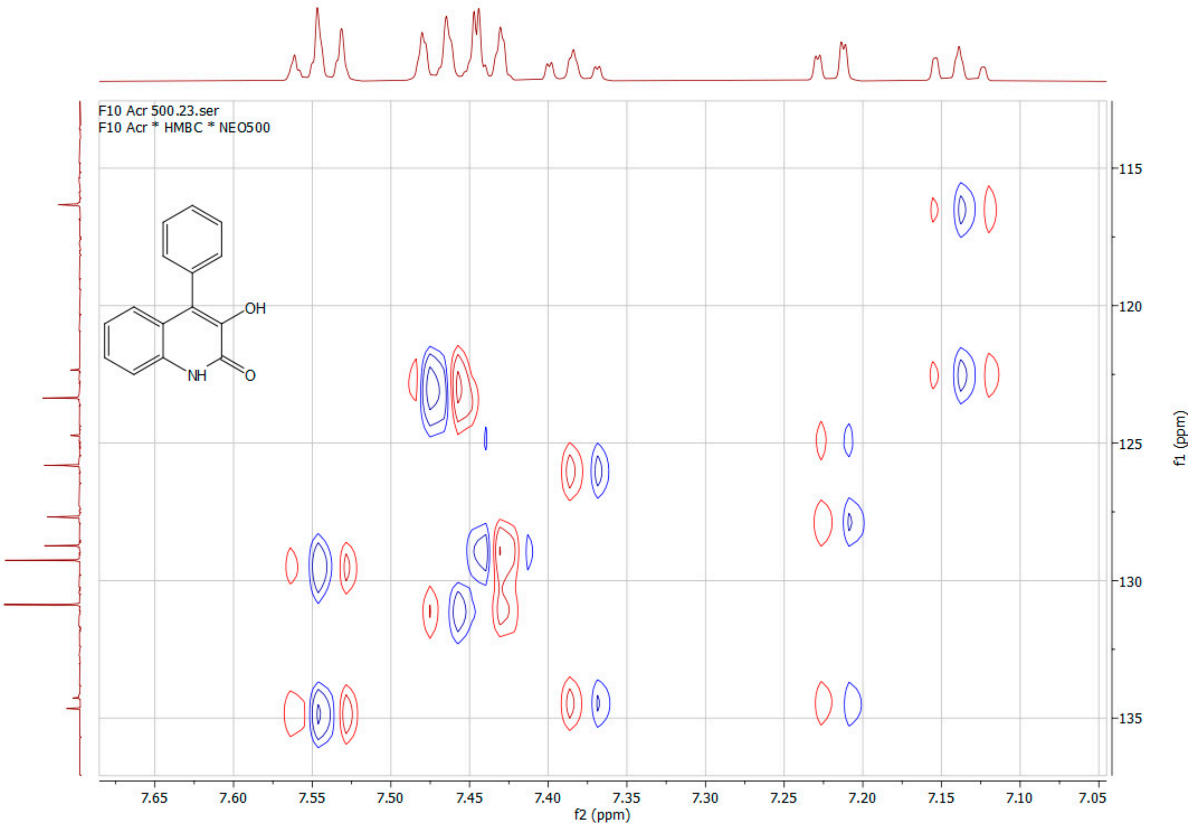

Figure S9: NOESY (500 MHz, acetone-d_6) of viridicatin.

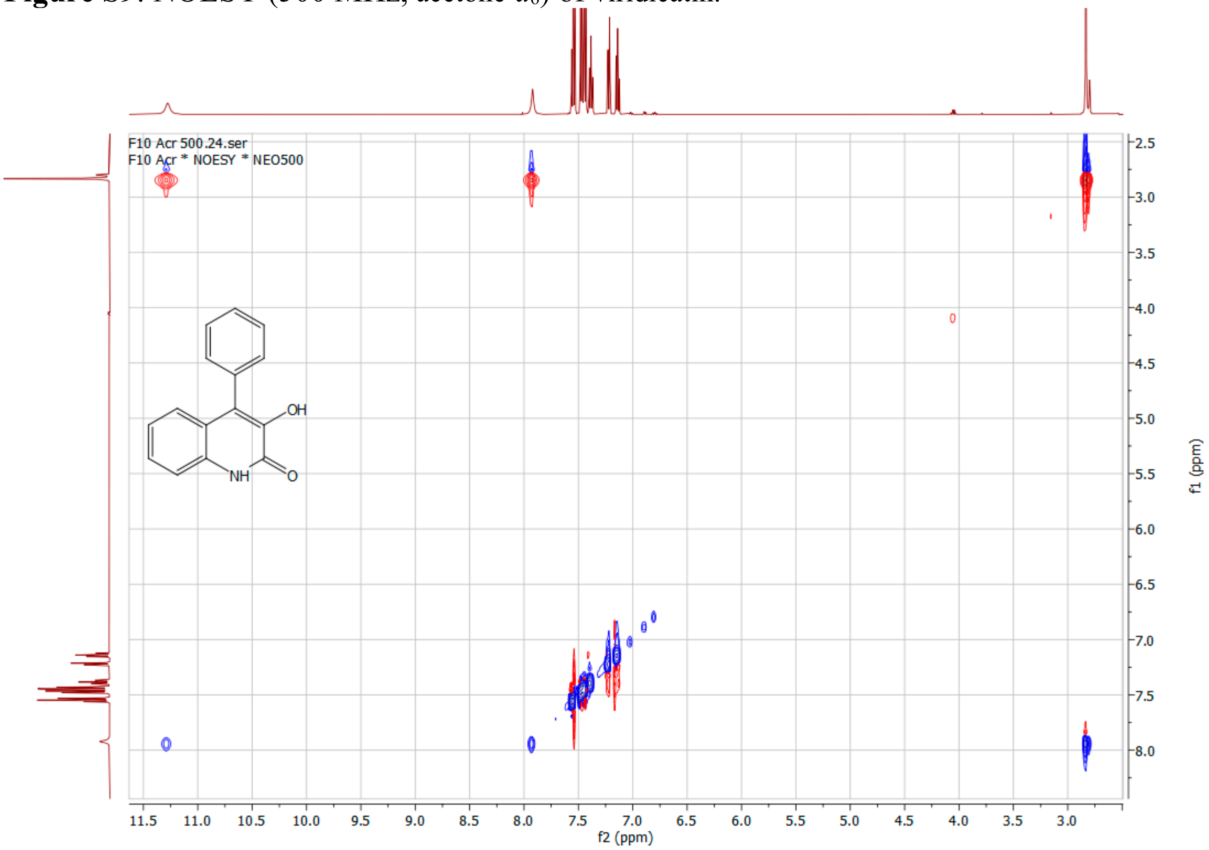

Figure S10. Mycoplasma testing of HMC-3 cultures before and after Plasmocin treatment.

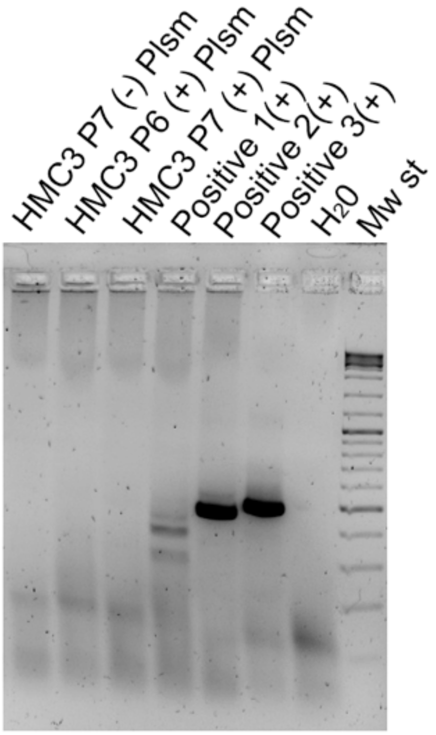

Representative endpoint PCR-based mycoplasma analysis was performed using the Mycoplasma PCR Detection Kit (Applied Biological Materials, abm, Cat. #G238). This conventional PCR assay detects more than 200 mycoplasma species/strains and yields a 450-bp amplicon in positive samples. PCR products were generated after 40 cycles, resolved by agarose gel electrophoresis, and visualized using SYBR Safe DNA Gel Stain (Invitrogen, Cat. #S33102). P indicates passage number before or after Plasmocin treatment. Three independent positive mycoplasma controls were included, one of which corresponded to the kit-provided positive control. H_2O was used as the no-template negative control, and MW st indicates the molecular weight standard. When indicated, after 6-7 passages cell cultures were routinely treated with Plasmocin according to the manufacturer's instructions for securing mycoplasma elimination (25 µg/ml for 2 weeks).

Figure S11. Viridicatin prevents the loss of viability induced by oxidative stress compared to basal viability in healthy patient-derived cells.

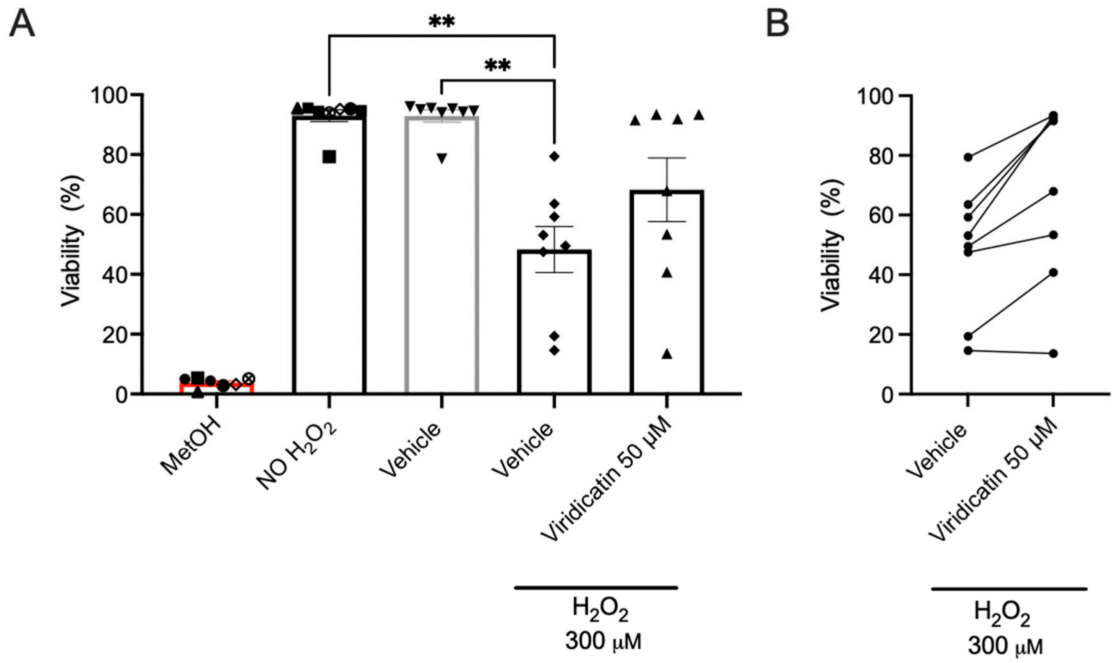

Cell viability was assessed under the indicated experimental conditions. No significant differences were observed between non-stimulated cells and their corresponding vehicle-treated controls. In contrast, exposure to H_2O_2 (300 µM) significantly reduced viability in vehicle-treated cells, whereas treatment with viridicatin (VIR, 50 µM) prevented this decrease. Treatment with methanol (MetOH) was performed as cell death positive control. Bars represent mean ± SEM, and symbols indicate individual patient-derived samples. $p < 0.01$. Data were analyzed using repeated-measures one-way ANOVA or the Friedman test, as appropriate, followed by multiple-comparisons testing. **B.** Paired representation of individual patient-derived samples exposed to H_2O_2 (300 µM) in the presence of vehicle or VIR (50 µM), highlighting the consistency of the viability-preserving effect across patients.

Supplementary Figure S12. Dynamic cross-correlation matrices (DCCM) from 100 ns MD simulations of viridicatin in complex with (A) KEAP1 (4IQK), (B) HO-1 (3CZY), and (C) NQO1 (1D4A). Blue regions indicate positively correlated residue motions; yellow/white regions indicate anti-correlated or uncorrelated motions.

Supplementary Figure S13. Principal component analysis (PCA) of MD trajectories for viridicatin in complex with (A) KEAP1 (4IQK), (B) HO-1 (3CZY), and (C) NQO1 (1D4A). Scatter plots show projections onto PC1 vs. PC2, PC3 vs. PC2, and PC1 vs. PC3. Scree plots show the proportion of variance explained by each eigenvalue. Color gradient from blue (early frames) to red (late frames) represents simulation time progression.

Figure S12 A: 4IQK Dccm

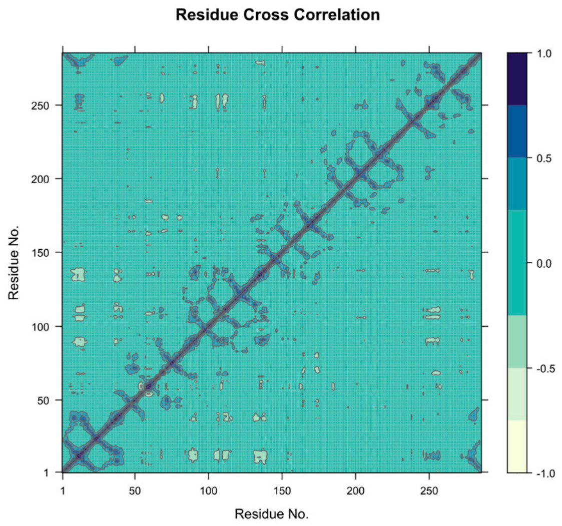

Figure S12B: 3CZY Dccm

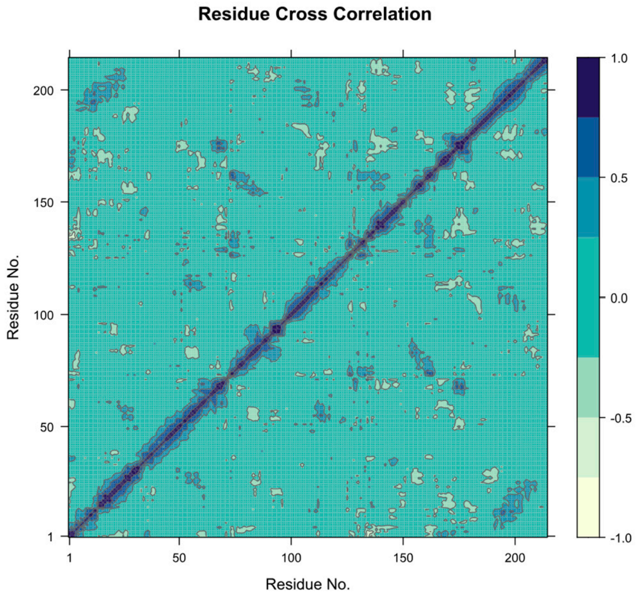

Figure S12C: 1D4A Dccm

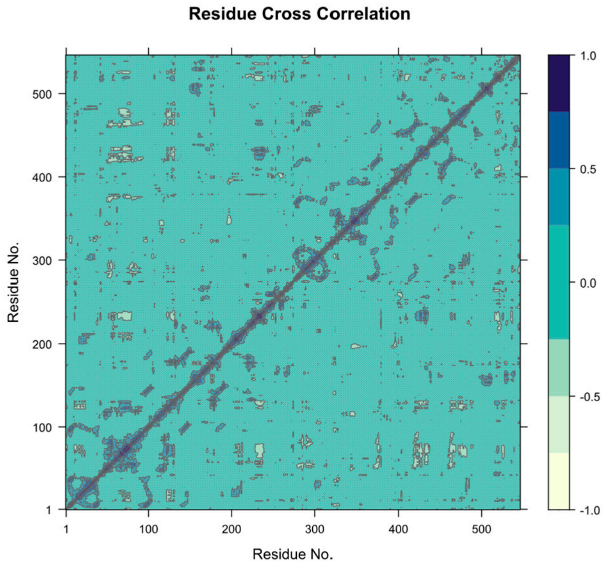

Figure 13A: 4IQK pcplot

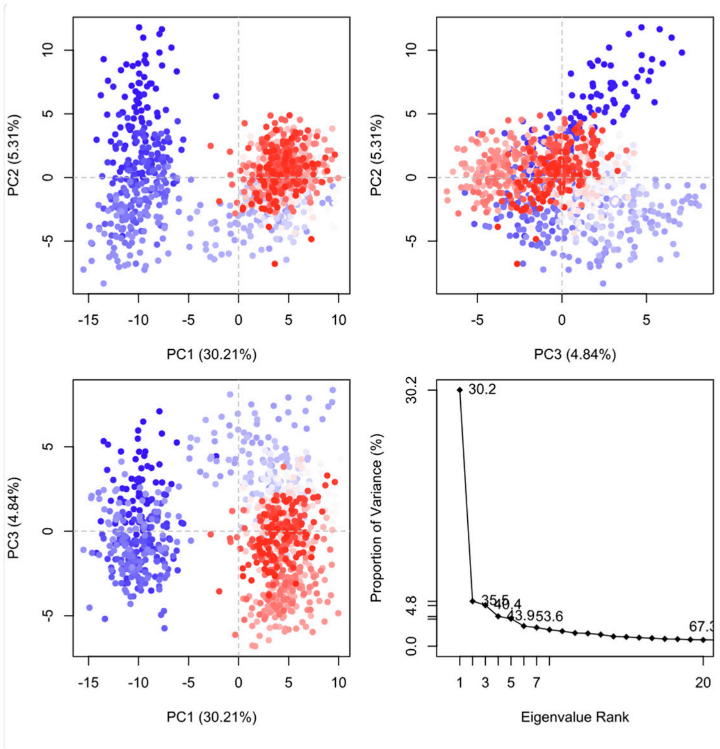

Figure 13B: 3CZY pcplot

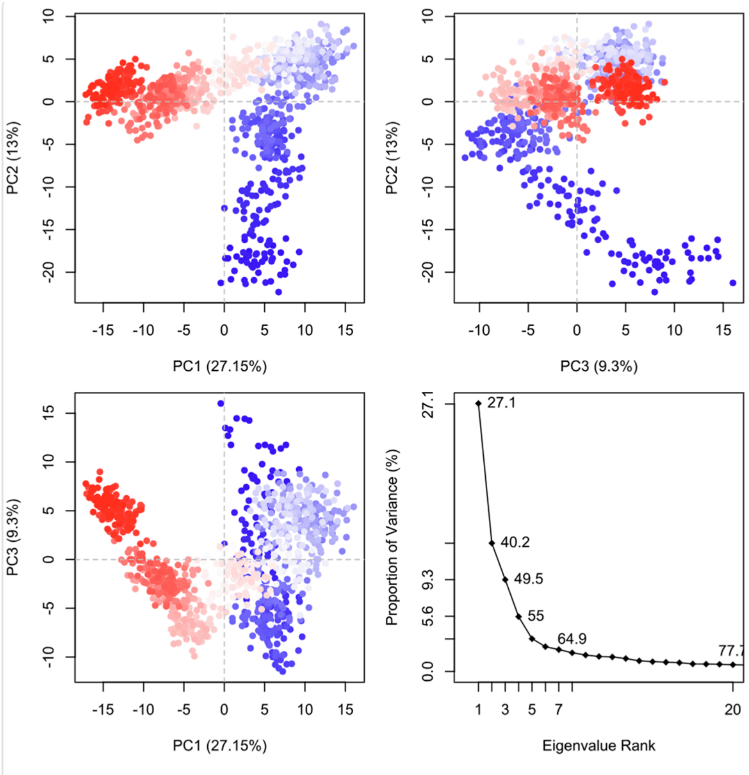

Figure 13C: 1D4A pcplot

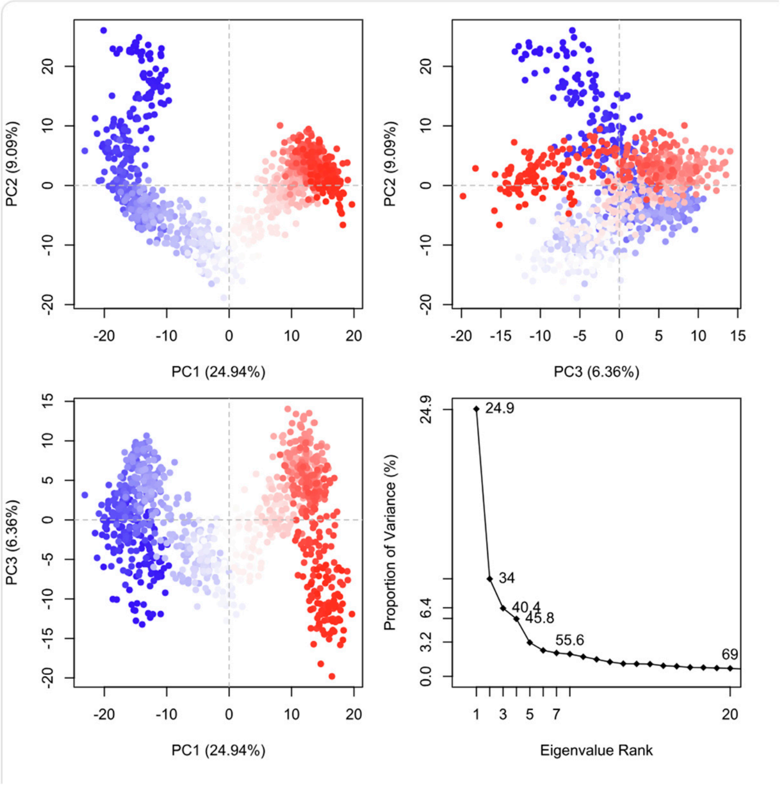

Supplement: Supplementary file 1 [file antioxidants-15-01089-s001.zip › antioxidants-4425537-supplementary.pdf]
